# SGLT2 inhibitors suppress epithelial–mesenchymal transition in podocytes under diabetic conditions *via* downregulating the IGF1R/PI3K pathway

**DOI:** 10.3389/fphar.2022.897167

**Published:** 2022-09-26

**Authors:** Ruixue Guo, Peipei Wang, Xuejun Zheng, Wen Cui, Jin Shang, Zhanzheng Zhao

**Affiliations:** ^1^ Department of Nephrology, The First Affiliated Hospital of Zhengzhou University, Zhengzhou, China; ^2^ Zhengzhou University, Zhengzhou, China; ^3^ Laboratory of Nephrology, The First Affiliated Hospital of Zhengzhou University, Zhengzhou, China; ^4^ Laboratory Animal Platform of Academy of Medical Sciences, Zhengzhou University, Zhengzhou, China

**Keywords:** diabetic nephropathy, sodium–glucose cotransporter-2 inhibitors, insulin-like growth factor-1 receptor, podocyte, epithelial–mesenchymal transition

## Abstract

Loss of podocyte is a characteristic pathological change of diabetic nephropathy (DN) which is associated with increased proteinuria. Many studies have shown that novel inhibitors of sodium–glucose cotransporter 2 (SGLT2-is), such as dapagliflozin, exert nephroprotective effect on delaying DN progression. However, the mechanisms underlying SGLT2-associated podocyte injury are still not fully elucidated. Here, we generated streptozotocin-induced DN models and treated them with dapagliflozin to explore the possible mechanisms underlying SGLT2 regulation. Compared to mice with DN, dapagliflozin-treated mice exhibited remission of pathological lesions, including glomerular sclerosis, thickening of the glomerular basement membrane (GBM), podocyte injury in the glomeruli, and decreased nephrotoxin levels accompanied by decreased SGLT2 expression. The mRNA expression profiles of these treated mice revealed the significance of the insulin-like growth factor-1 receptor (IGF1R)/PI3K regulatory axis in glomerular injury. KEGG analysis confirmed that the phosphatidylinositol signaling system and insulin signaling pathway were enriched. Western blotting showed that SGLT2-is inhibited the increase of mesenchymal markers (α-SMA, SNAI-1, and ZEB2) and the loss of podocyte markers (nephrin and E-cad). Additionally, SGLT2, IGF1R, phosphorylated PI3K, α-SMA, SNAI-1, and ZEB2 protein levels were increased in high glucose-stimulated human podocytes (HPC) and significantly decreased in dapagliflozin-treated (50 nM and 100 nM) or OSI-906-treated (inhibitor of IGF1R, 60 nM) groups. However, the use of both inhibitors did not enhance this protective effect. Next, we analyzed urine and plasma samples from a cohort consisting of 13 healthy people and 19 DN patients who were administered with (*n* = 9) or without (*n* = 10) SGLT2 inhibitors. ELISA results showed decreased circulating levels of IGF1 and IGF2 in SGLT2-is-treated DN patients compared with DN patients. Taken together, our study reported the key role of SGLT2/IGF1R/PI3K signaling in regulating podocyte epithelial–mesenchymal transition (EMT). Modulating IGF1R expression may be a novel approach for DN therapy.

## 1 Introduction

Diabetic nephropathy (DN) is associated with increasing incidence (Zhang et al., 2010; Ogurtsova et al., 2017) and poor prognosis (Alicic et al., 2017; Cheng et al., 2021). Patients identified as having diabetes mellitus (DM) exhibit higher filtration and reabsorption of glucose in the kidney, which is associated with the upregulated expression of sodium–glucose cotransporter 2 (SGLT2) in the proximal convoluted tubules (Vallon and Verma, 2021). This excessive increase in SGLT2 expression further promotes the accumulation of glucose in the host, which forms a vicious cycle and causes lasting kidney damage. The glomerular pathology that occurs secondary to diabetes is characterized by the thickening of the glomerular basement membrane (GBM) and podocyte injury (Rabkin, 2003). In the early stage of DN, podocytes can become hypertrophic to respond to the tractive effects of the thickened GBM. However, constant stimulation ultimately leads to the dissociation of the foot process and basement membrane, as well as the loss of cell proliferation ability (Xu et al., 2010). Podocytes are the last and most critical components of the glomerular filtration barrier, and the loss of this function is closely associated with the onset of proteinuria (Mundel and Reiser, 2010). According to reports, an elevated level of proteinuria is positively correlated with adverse renal outcomes (Lambers Heerspink and Gansevoort, 2015). Therefore, blocking SGLT2-mediated glucose transport in DN, even DM, helps attenuate the progression of proteinuria.

Recently, numerous studies reported the reno-protective effects of SGLT2-is, a new hypoglycemic drug, such as dapagliflozin, in treating DN and its cardiovascular complications (Kovesdy et al., 2022; Liu et al., 2022). These beneficial effects can be attributed to the effects of SGLT2-is on the decreased regulation of glomerular filtration and proteinuria (Korbut et al., 2020; Ravindran and Munusamy, 2022). For instance, dapagliflozin dilates the efferent arteriole, reduces intravascular pressure, and alleviates shear force injury to the filtration barrier (van Bommel et al., 2020). In BTBR ob/ob mice, empagliflozin decreased the expression of SGLT2 in podocytes and further improved the microvascular endothelial ultrastructure by inhibiting the secretion of podocyte-derived VEGF-A (Locatelli et al., 2022). Cassis et al. (2018) reported a high level of SGLT2 in protein-overloaded human podocytes (HPC), and inhibition of SGLT2 could help normalize podocyte cytoskeleton and function. However, much evidence regarding the possible molecular mechanisms by which SGLT2-is affect glomeruli, especially in podocytes, is still needed.

For this purpose, we established SGLT2-is-treated diabetic models and found that kidney injury caused by SGLT2 upregulation was mediated by insulin-like growth factor 1 receptor (IGF1R). In parallel, we reconfirmed the causal relationship between SGLT2 and IGF1R in HPC cultured with high glucose. SGLT2/IGF1R signaling mainly mediated EMT in podocytes. Moreover, efficacy evaluations in DN patients revealed the direct effect of SGLT2 on IGF1R ligands. All these results showed that inhibition of IGF1R might be an alternative approach to normalize the function of podocytes and reduce proteinuria, which provided a new idea for DN therapy.

## 2 Materials and methods

### 2.1 Management of animal models

A total of 18 six-week-old specific pathogen-free (SPF) male C57BL6J mice were purchased from Huafukang Laboratory Animals Center (Beijing, China) and were randomly divided into Con (*n* = 6), DN (*n* = 6), and dapagliflozin-treated DN groups (DA, *n* = 6). Prior to our experiment, the mice were allowed to adapt to the suitable laboratory conditions (room temperature of 22°C; 12 h light/dark cycle) for 2 weeks. Meanwhile, the mice designed as diabetes models (the DN and DA groups) were given a high-fat diet (fat content: 60%). In the eighth week, the mice that were assigned to diabetic groups were intraperitoneally injected with streptozotocin (50 mg/kg/d, Sigma), and the mice in the Con group were given an equal volume of saline for five consecutive days. Diabetic mice were defined as having a blood glucose (BG) level >=17.6 mmol/L in the tenth week. In the subsequent 4 weeks, dapagliflozin was dissolved in sodium hydroxymethyl cellulose and delivered to the mice (DA group) by oral gavage (1 mg/kg/d). The rest of the mice (DN and Con groups) were treated with equal amounts of normal saline. All the mice were euthanized at 18th weeks. The Institutional Review Board of the Experimental Animal Center of Zhengzhou University approved our animal experiment (ZZU-LAC20210402 [09]).

### 2.2 Measurement of laboratory parameters

Laboratory parameters such as body weight (BW), BG, and protein/creatinine ratio (PCR) were recorded in the 8th, 10th, 14th, and 18th weeks. Mouse tails were punctured to collect blood for BG measurements. The PCR was measured on a platform in our hospital laboratory department. In addition, we obtained the jugular venous blood to measure the biochemical indexes. Biochemical detection kits for serum creatinine (50 ul/well, sarcosine oxidase method) (Liang et al., 2017) and blood urea nitrogen (BUN) (10 ul/well) were obtained from Shanghai Meilian Biotechnology Co., Ltd., (Shanghai, China).

### 2.3 Histological analysis of the animal renal cortex

After blood collection, we immediately blanched the mouse kidney by perfusing the left ventricle using normal saline (NS). When both kidneys were white, we resected and isolated sections of the renal cortex from the medulla and placed them in a -80°C environment for subsequent mRNA sequencing and WB analysis. In addition, another small piece of the cortex was collected and stored in fixative to perform TEM as previously described (Veron et al., 2011). The remaining renal cortex was incubated in 4% paraformaldehyde (PFA), embedded in paraffin for 48 h, and finally processed into 4-µm-thick sections for hematoxylin/eosin (HE), periodic acid–Schiff’s reagent (PAS), Masson (Veron et al., 2021) and immunohistochemical (IHC) staining. IHC of frozen kidney sections was performed using primary antibodies against SGLT2 (1:100), IGF1R (1:1000), IGF1 (1:500), and collagen IV (1:500)) (Veron et al., 2014). As previously described, we also focused on SGLT2 expression in glomeruli using immunofluorescence staining (Kato et al., 2006).

### 2.4 mRNA assay

mRNA sequencing was performed on the renal cortex of all mice (*n* = 18). Total RNA was isolated following the instructions of the RNAeasy Mini Kit (Qiagen, Germany). Poly-T oligo-conjugated magnetic beads were used to purify mRNA molecules containing poly-A. We first fragmented the mRNA under high temperature and then performed reverse transcription to generate a cDNA library. The Qubit® 2.0 Fluorometer (Life Technologies, United States) and Agilent 2100 bioanalyzer (Agilent Technologies, United States) were used to confirm the gene concentration. Clusters of mRNAs were further sequenced on an Illumina NovaSeq 6000 (Illumina, United States). Total RNA isolation, cDNA library establishment, and Illumina sequencing were all conducted by Sinotech Genomics Co., Ltd., (Shanghai, China). The RNA concentration was assessed using FPKM, which is defined as fragments per kilobase of exon per million reads mapped. The fragments within each gene were counted using StringTie software and then normalized using the TMM algorithm. Differentially expressed genes (DEGs) were selected using the edgeR R package. PCA/heatmap/volcano plots visualized the expression levels of the DEGs among the three groups. SangerBox was used to analyze Kyoto Encyclopedia of Genes and Genomes (KEGG) pathway enrichment. The construction of a protein–protein interaction (PPI) with IGF1R as the core was performed using the following steps: first, Cytoscape software was used to identify the top 20 core genes from 1957 DEGs (|log2(FC)| value >1 and p-adjusted value/q < 0.05), which were coexpressed in Con vs. DN and DN vs. DA comparisons. The results suggested that the IGF1R/PI3K pathway was the most significantly modulated by SGLT2. Second, genes with combined scores larger than 0.9 with IGF1R were selected for PPI in analysis using the STRING website (https://string-db.org/).

### 2.5 Cell culture and inhibitor use

Conditionally, immortalized HPC referred to in our study were donated by Shandong University. HPC and human glomerular endothelial cells (HGRECs) were normally expanded in 1640 medium supplemented with 1% penicillin–streptomycin, 10% FBS, and 5.6 mM glucose. Mesangial cells (HMCs) and HK-2 cells (a kind of tubular epithelial cell) were cultured in DMEM. To explore the optimal experimental conditions, 2*10^6^ cells were seeded in six-well plates, serum-starved for 24 h, and stimulated with 20 mM or 40 mM glucose for 24 h (cell density: 50%) or 48 h (cell density: 30%). Inhibitor-treated groups were set with different doses of inhibitors: dapagliflozin (Macklin, 461432-26-8; 50 nM, 100 nM, or 250 nM), OSI-906 (IGF1R antagonist, MedChemExpress, HY-10191; 30 nM or 60 nM), and Da+OSI-906 (50 nM + 60 nM).

### 2.6 Western blotting

The extracts from the renal cortex or cultured cells were incubated with antibodies against SGLT2 (ab37296, 2 ug/ml), IGF1R (ab182408, 1/2000), PI3K-p85 alpha (AF6241, 1/2000), p-PI3K-p85 (Tyr458/p55Try199) (AF3242, 1:1000), α-SMA (ab21027, 1/400), nephrin (ab58968, 1/1000), E-cadherin (60335-1-AP, 1:1000), SNAI-1 (26183-1-AP, 1:1000), and ZEB2 (14026-1-AP, 1:1000). Protein expression *in vitro* and *in vivo* was quantified using ImageJ (https://imagej.nih.gov/ij/) and processed using Prism 8 software.

### 2.7 Human specimen collection

Study design: from 2018 to 2020, urine and plasma samples from 1030 patients who were diagnosed with DN were collected and stored in the Biobank of The First Affiliated Hospital of Zhengzhou University. After strict screening criteria described in the following paragraph, 10 patients with DN and 9 SGLT2-is-treated DN patients were ultimately included in our study. Thirteen-year-old/sex-matched healthy people were recruited as controls from the Physical Examination Center of our hospital.

Inclusion criteria: there were two evaluative criteria for the control groups: 1) with normal laboratory tests including kidney and liver function tests and routine blood examination and 2) no drug treatment. The diagnostic criteria of DN were in accordance with the 2012 KDIGO (Levey et al., 2011). Based on the former requirement, the SGLT2-is-treated group should have completed taking SGLT2-is (i.e., dapagliflozin, empagliflozin, and canagliflozin) for more than 1 month.

Exclusion criteria: subjects who suffered cancer, hepatic diseases, endocrine system diseases (i.e., pituitary tumor), and a combination of other urinary system diseases (e.g., ANCA-associated vasculitis with renal damage, IgA nephropathy, membranous nephropathy, and end-stage renal disease) or who received dialysis-related treatments were excluded. Notably, we also excluded DN patients who were treated with exogenous insulin.

Others: the use of these patients’ specimens was approved by the Institutional Review Board of The First Affiliated Hospital of Zhengzhou University (2019-KY-361) and followed the Declaration of Helsinki. All the participants signed written informed consent forms.

The levels of IGF1 and IGF2 in all obtained specimens were tested using ELISA kits according to the manufacturer’s instructions.

### 2.8 Statistical analysis

The statistical significance of BG, BW, and PCR among the three groups was calculated using two-way ANOVA followed by Tukey’s *post hoc* method, while one-way ANOVA was used for the other statistical analysis.

## 3 Results

### 3.1 Sodium–glucose cotransporter 2-is attenuated the pathological progression and improved laboratory parameters in mice with diabetic nephropathy

**FIGURE 1:**
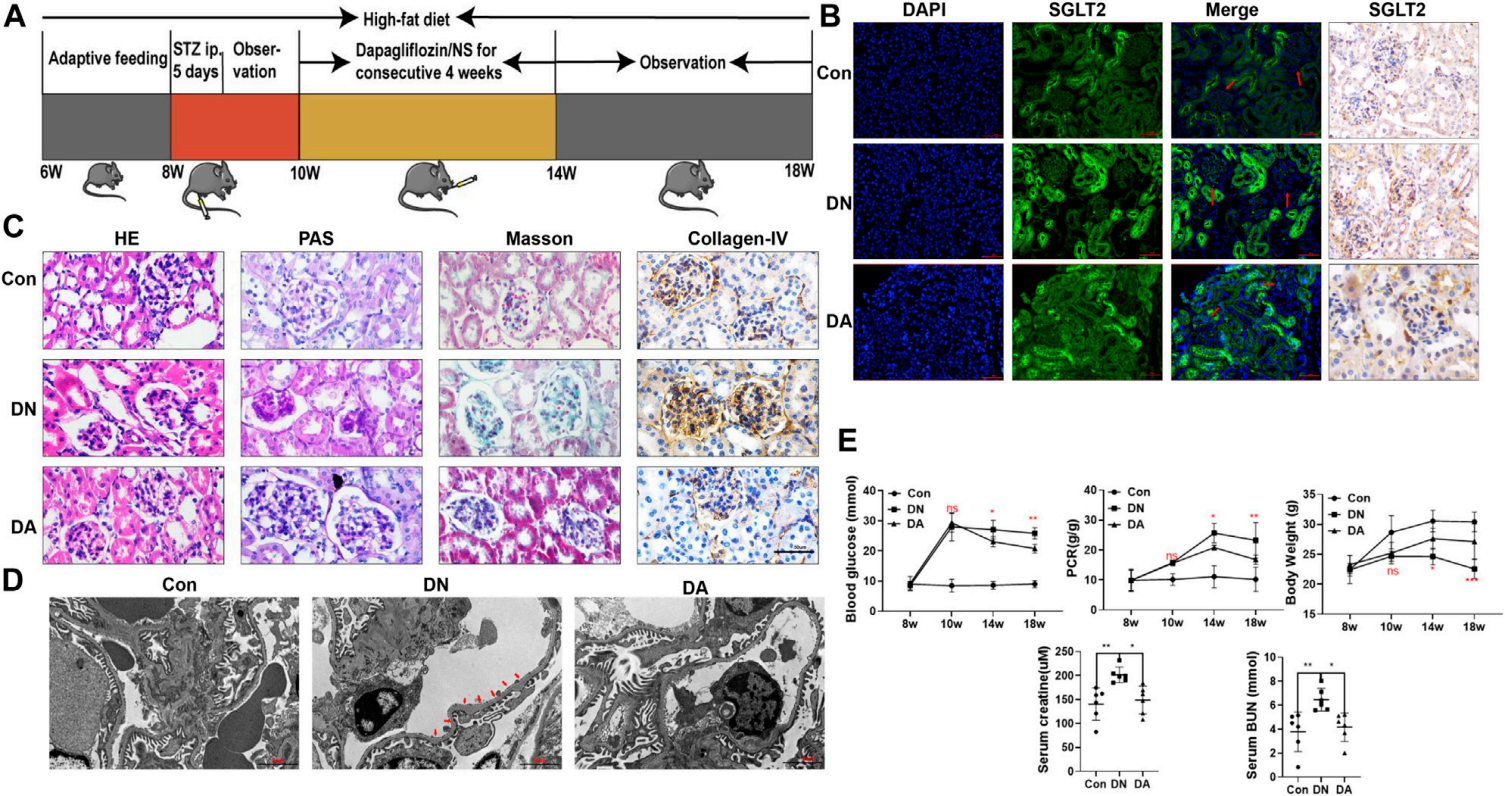
Dapagliflozin attenuated STZ-induced diabetic kidney injury. **(A)** Flowchart of the animal experiment (*n* = 6/group). **(B)** IF and IHC staining of SGLT2. **(C)** Pathological staining: light micrographs of HE, PAS, and Masson staining and IHC of collagen IV. **(D)** Representative electron micrographs of glomeruli. Areas of basement membrane thickening and podocyte injury are indicated by red arrows. **(E)** Statistical significance of BG levels, BW, and PCR among the three groups in the 8th, 10th, 14th, and 18th weeks was performed using two-way ANOVA, and Tukey’s algorithm for subsequent multiple comparisons between two groups. In parallel, one-way ANOVA was performed for SCr and BUN levels. STZ: streptozotocin; Con: control group; DN: diabetic nephropathy; DA: dapagliflozin-treated DN group: NS: normal saline; SGLT2: sodium–glucose cotransporter 2; IF: immunofluorescent staining; IHC: immunohistochemical staining; one-way ANOVA: one-way analysis of variance; BG: blood glucose; BW: body weight; PCR: urinary total protein to creatinine ratio; SCr: serum creatinine; BUN: blood urea nitrogen.

To evaluate the reno-protective effect of SGLT2 inhibitors on kidney pathology and function in diabetic mice, we established Con, DN, and dapagliflozin-treated groups (Figure 1A). First, IHC and IF staining both indicated a wider distribution of SGLT2 in the glomeruli of the DN group than in the Con or DA group (Figure 1B, Supplementary Figure S1). The diabetic mice exhibited smaller and sclerotic glomeruli with an expanded mesangial matrix, increased glycogen deposition, and obvious collagen fiber after 2 months, while all these lesions were improved in the dapagliflozin-treated mice with DN (Figure 1C, Supplementary Figure S2). Measurement of the GBM in the DN group showed an irregular thickening and missing podocyte processes, but no significant differences were observed between the DA group and Con group (Figure 1D and Supplementary Figure S3). Laboratory variables, including BG levels, PCR, Scr levels, and BUN levels were lower and BW was higher in the DA group than in the DN group (Figure 1E). These results suggested that suppression of SGLT2 could alleviate glucose-induced DN progression.

### 3.2 mRNA profile in dapagliflozin-treated mice

**FIGURE 2:**
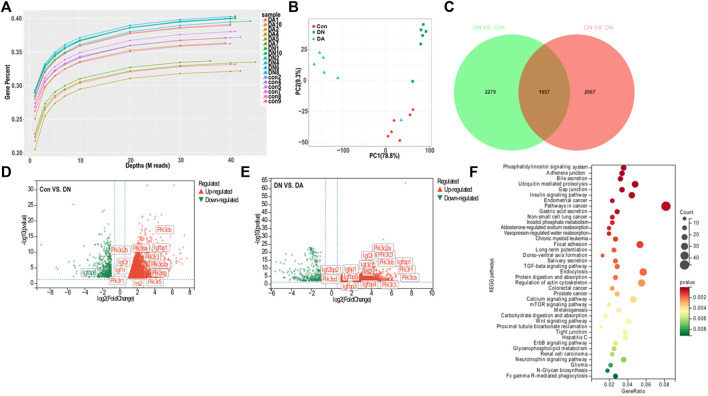
Gene expression profile of dapagliflozin-treated diabetic models. **(A)** Gene saturation analysis. **(B)** PCA plot within groups. The values of principal components 1 and 2 were 78.8% and 9.3%, respectively. **(C)** Venn diagram of all the DEGs between Con vs. DN and DN vs. DA. Volcano plots of the DEGs between DN and Con **(D)** and between DN and DA **(E)**. Red dots: upregulated in DN; green dots: downregulated in DN. **(F)** KEGG analysis revealed enriched pathways modulated by the DEGs among the three groups (p-adjusted<0.05). X-axis: gene-ratio = genes with significance/all involved genes; circle color represents significance, and circle size represents counts of the DEGs. PCA: principal component analysis; IGF1R: insulin-like growth factor-1 receptor; DEGs: differentially expressed genes (p/q < 0.05 and |log2(FC)| >1).

We first explored the molecular effects of dapagliflozin on the basis of transcriptomics. Gene-depth curves showed that the depth of the estimated genes approached saturation in each sample (Figure 2A). The PCA plot indicated a relatively concentrated distribution within groups (Figure 2B). As shown in the Venn diagram, 1957 genes were shared between the Con vs. DN and DA vs. DN comparisons (p/q < 0.05 and |log2(FC)|>1, Figure 2C, Supplementary Figure S4). The expression profiles of the 1957 genes among the three groups were shown in a heatmap (Supplementary Figure S5, Supplementary Table S1). The volcano plot showed the upregulated and downregulated genes in the DN vs. DA and Con vs. DN comparisons (Figures 2D,E). After KEGG enrichment analysis of 1957 DEGs, 39 signaling pathways, including inositol phosphate metabolism, phosphatidylinositol signaling system, insulin signaling pathway, mTOR signaling pathway, and TGF-β signaling pathway, remained significantly associated with dapagliflozin use (p-adjusted<0.05, Figure 2F).

### 3.3 Upregulation of sodium–glucose cotransporter 2 caused glomerular injury mainly *via* the insulin-like growth factor 1 receptor/PI3K pathway

**FIGURE 3:**
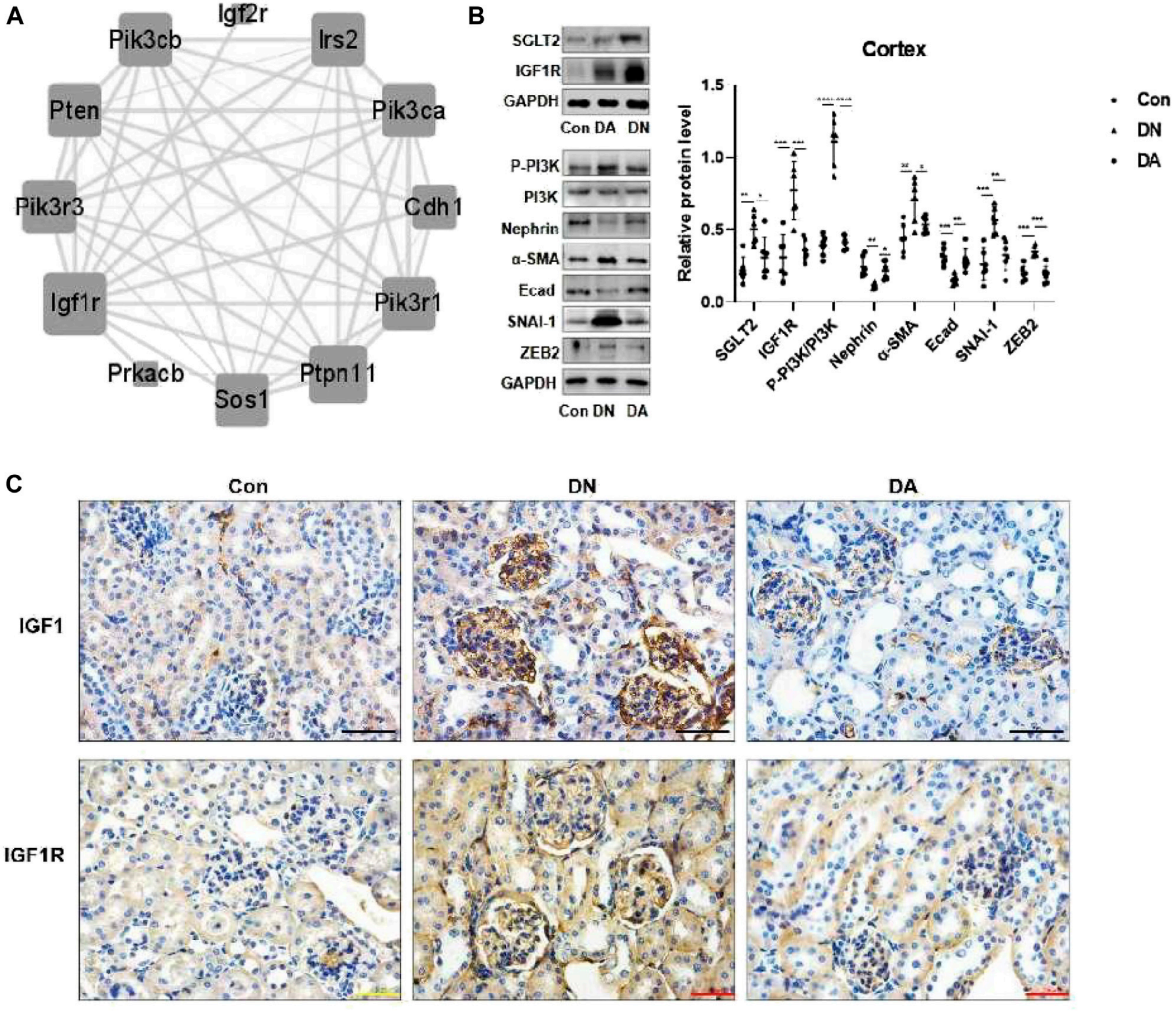
In DN models, upregulation of SGLT2 activated IGF1R/PI3K signaling. **(A)** IGF1R-centered PPI network (combined score>0.9). **(B)** Protein expression levels of SGLT2, IGF1R, phosphorylated PI3K, and EMT markers were analyzed using Western blotting. The data were analyzed by ANOVA with Tukey’s post hoc test. **(C)** IHC images of IGF1 and IGF1R. PPI: protein–protein interaction; *p < 0.05, **p < 0.01.

Through six algorithms provided using Cytoscape software, we observed a core status of IGF1R among the 1957 DEGs (Table 1). Moreover, PI3K signaling exhibited strong crosstalk with IGF1R (Figure 3A; Supplementary Figure S6). Compared with those in the DA or Con group, the expression levels of factors related to the IGF system, PI3K subunits, and markers of epithelial–interstitial trans-differentiation (EMT) were all increased in the DN group (Supplementary Figure S7). A study reported that IGF1-receptor expression was significantly increased in diabetic models with kidney damage, and this receptor partly activated downstream PI3K signaling in a phosphatase-dependent manner, ultimately leading to the accumulation of toxic substances in podocytes (Korbut et al., 2020). Therefore, we proposed the hypothesis that inhibition of SGLT2/IGF1R/PI3K signaling plays an important role in protecting against DN progression.

**TABLE 1:** Using Cytoscape to determine the top 20 hub genes from 1957 DEGs.

Scorename method	Closeness	Degree	EPC	MCC	MNC	Radiality
Pten	766.05	148	156.627	2.2026E+11	145	7.708313115
Cdh1	726.88333	121	146.143	—	120	7.580855815
Pik3ca	713.9	114	151.778	2.27117E+11	114	7.53963828
Mapk1	703.4	93	137.49	—	93	7.523785382
Ep300	700.68333	105	131.263	—	102	7.493981934
Cltc	700.08333	84	—	—	80	7.526955962
Pik3r1	694.6	94	148.31	2.26976E+11	93	7.487006659
Nina	694.1	81	124.133	—	77	7.501591325
Igfir	690.43333	75	138.306	19488251184	75	7.497152514
Atm	685.46667	79	—	—	77	7.473690225
Erbb4	684.36667	78	134.207	2.2702E+11	77	7.467983181
Smad4	683.83333	82	132.157	—	82	7.457203211
Arf6	679.8	84	123.035	—	81	7.437545617
Yes1	679.55	83	132.845	1.22932E+11	78	7.438179733
Htt	677.56667	—	—	—	—	7.465446718
Prkacb	672.75	77	—	—	75	7.417253908
Pik3cb	670.55	79	133.311	2.26923E+11	79	—
Traf6	670.25	—	—	—	—	7.421058603
Ptk2b	669.78333	83	129.637	2.00279E+11	81	—
Lrrk2	669.75	—	—	—	—	7.43564327
Kdr	—	78	129.232	1.82683E+11	75	—
Pik3r3	—	76	132.562	2.26932E+11	74	—
Ptpn11	—	74	130.379	2.27085E+11	71	—
Erbb3	—	—	131.252	2.27017E+11	—	7.410278633
Map2k1	—	—	126.476	10276488566	—	7.409644517
Frk	—	—	124.383	1.0408E+11	—	—
Sos1	—	—	—	2.26385E+11	—	—
Sos2	—	—	—	2.26374E+11	—	—
Cbl	—	—	—	2.14613E+11	—	—
Flt1	—	—	—	1.75403E+11	—	—
Jak1	—	—	—	28052807594	—	—
Lyn	—	—	—	16332161318	—	—

To verify the effects of dapagliflozin on IGF1R signals, we measured the expression of relevant proteins in the renal cortex in response to high-glucose simulation. Western blotting revealed that dapagliflozin decreased the protein levels of IGF1R and phosphorylated PI3K but prevented the loss of nephrin, a marker of podocytes, and E-cad. Moreover, diabetic mice treated with dapagliflozin exhibited a reduction in the expression of α-SMA, SNAI-1, and ZEB2, which were implicated in EMT (Figure 3B). IHC staining revealed a wide distribution of IGF1 and IGF1R expression in DN glomeruli, compared with dapagliflozin-treated models (Figure 3C, Supplementary Figure S8).

### 3.4 Inhibition of the sodium–glucose cotransporter 2/insulin-like growth factor 1 receptor pathway reduced epithelial–interstitial trans-differentiation in high-glucose-stimulated podocytes

**FIGURE 4:**
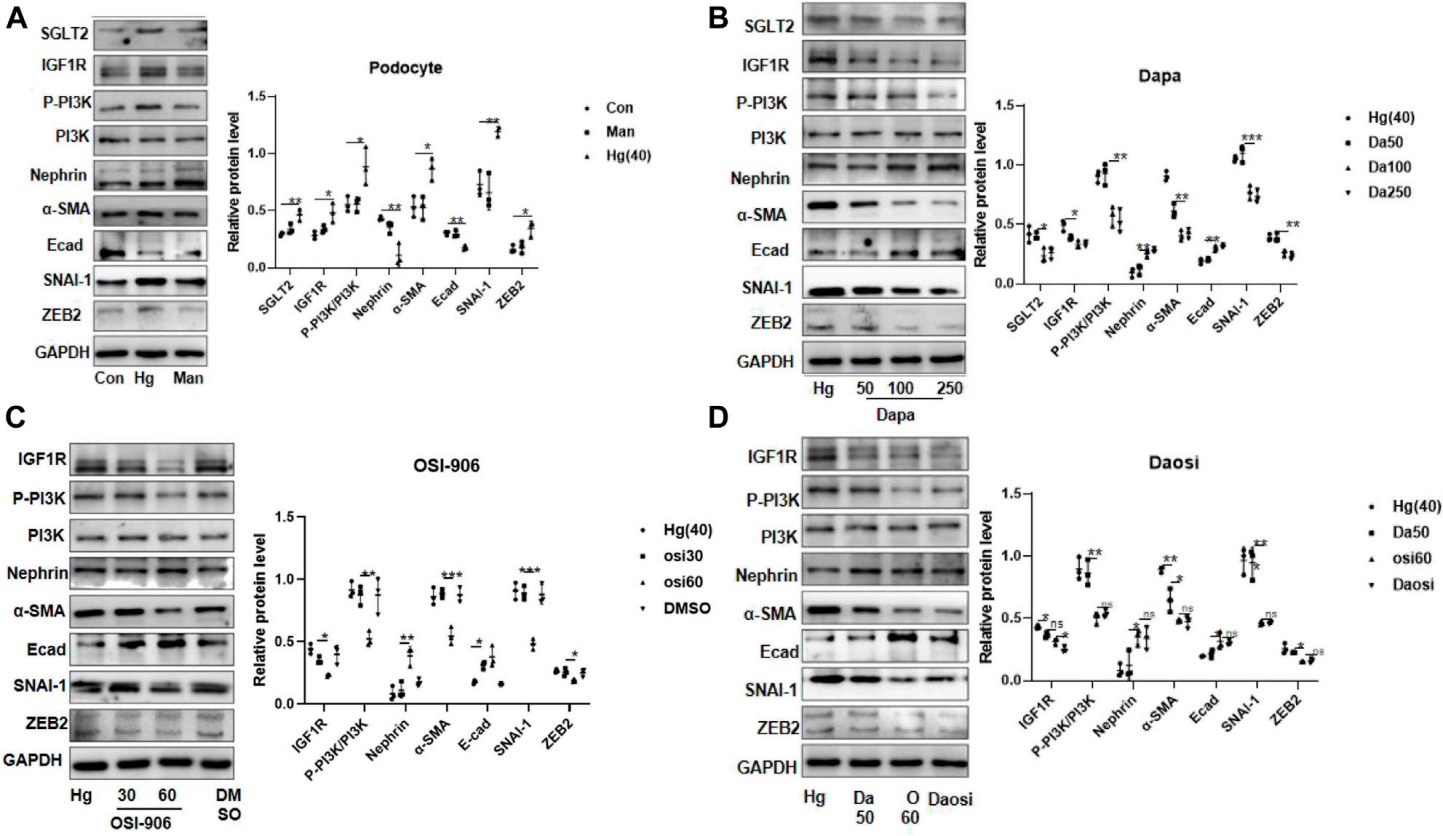
SGLT2 directly regulated podocyte EMT *via* IGF1R/PI3K signaling. **(A)** Representative WB images and densitometric analysis of the expression of SGLT2, IGF1R, phosphorylated PI3K, and EMT markers in high-glucose-stimulated podocytes (Hg: 40 nM, 24 h). **(B)** Podocytes treated with different doses of dapagliflozin (50 nM, 100 nM, and 250 nM) and **(C)** OSI-906 (30 nM and 60 nM). **(D)** Combination of OSI60 and Da50. EMT: epithelial–mesenchymal transition. The data were analyzed by ANOVA with Tukey’s post hoc test. N = 3; *p < 0.05, **p < 0.01, ***p < 0.001, ****p < 0.001.

After 24 h or 48 h of stimulation under high-glucose conditions (20 mM and 40 mM), the protein expression of IGF1R in HPC or HGREC was dramatically increased, but the change in the expression of the latter was mainly attributed to the effects of hyper-osmosis. In addition, we did not observe obvious glucose-induced alterations in IGF1R protein expression in HMC or HK-2 cells (Supplementary Figure S9). Therefore, we chose HPC for further analysis. HPC were exposed to 40 mM glucose for 24 h. Consistent with the results in diabetic mice, we observed similar trends of protein expression in the high-glucose-stimulated podocytes (Figure 4A). Interestingly, dapagliflozin-treated podocytes presented a dose-dependent reduction in the levels of IGF1R, phosphorylated PI3K, α-SMA, SNAI-1, and ZEB2 and an upregulation of the levels of nephrin and E-cad (Figure 4B). We further cultured high-glucose-stimulated podocytes with OSI-906 (30 or 60 nM), a selective IGF1R inhibitor, to elucidate the relationship between IGF1R and PI3K. As expected, OSI-906 downregulated phosphorylated PI3K, α-SMA, SNAI-1, and ZEB2 and upregulated nephrin and E-cad levels in a dose-dependent manner (Figure 4C). Additional cotreatment with OSI-906 (60 nM) and dapagliflozin (50 nM) was performed to explore the favorable effect on podocyte repair. The combined process failed to enhance the reno-protective effects (Figure 4D). Altogether, our results demonstrated a critical role of SGLT2/IGF1R signaling in suppressing podocyte injury.

### 3.5 Sodium–glucose cotransporter 2-is alleviated the progression of diabetic nephropathy accompanied by decreased circulation and excretion of insulin-like growth factor-1 levels

**FIGURE 5:**
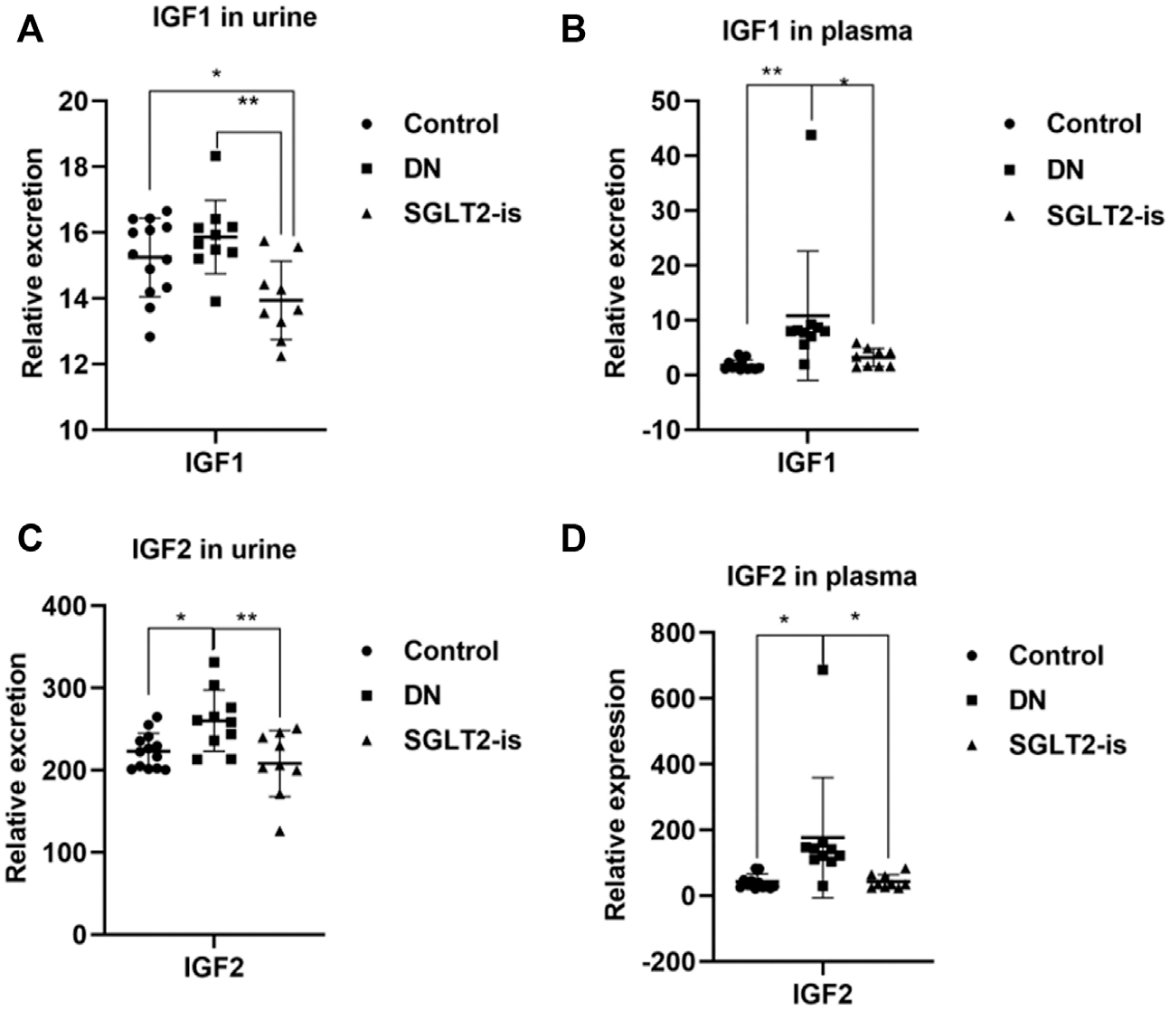
IGF1 and IGF2 expression in DN patients with or without SGLT2-is use. Excreted and circulating levels of IGF1 **(A,B)** and **(C,D)** IGF2 in human urine and plasma. Healthy controls: *n* = 13; DN patients without SGLT2-is use: *n* = 10; DN patients with SGLT2-is use: *n* = 9. The data were analyzed by ANOVA with Tukey’s post hoc test. * *p* < 0.05, **p < 0.01.

After a strict screening process, a total of 19 volunteers with DN who were taking SGLT2 inhibitors (*n* = 9) or not (*n* = 10) were enrolled. The statistical significance of the biochemical variables of all the included participants is shown in Supplementary Table S2. We found increased circulation of IGF1 and IGF2 in the patients with DN compared with the healthy controls, which could be reversed after SGLT2-is use (Figure 5A–D). This suggested that SGLT2-is might inhibit hepatic synthesis of IGF1 and IGF2, thereby reducing the secretion and excretion of IGF1R ligands.

## 4 Discussion

**FIGURE 6:**
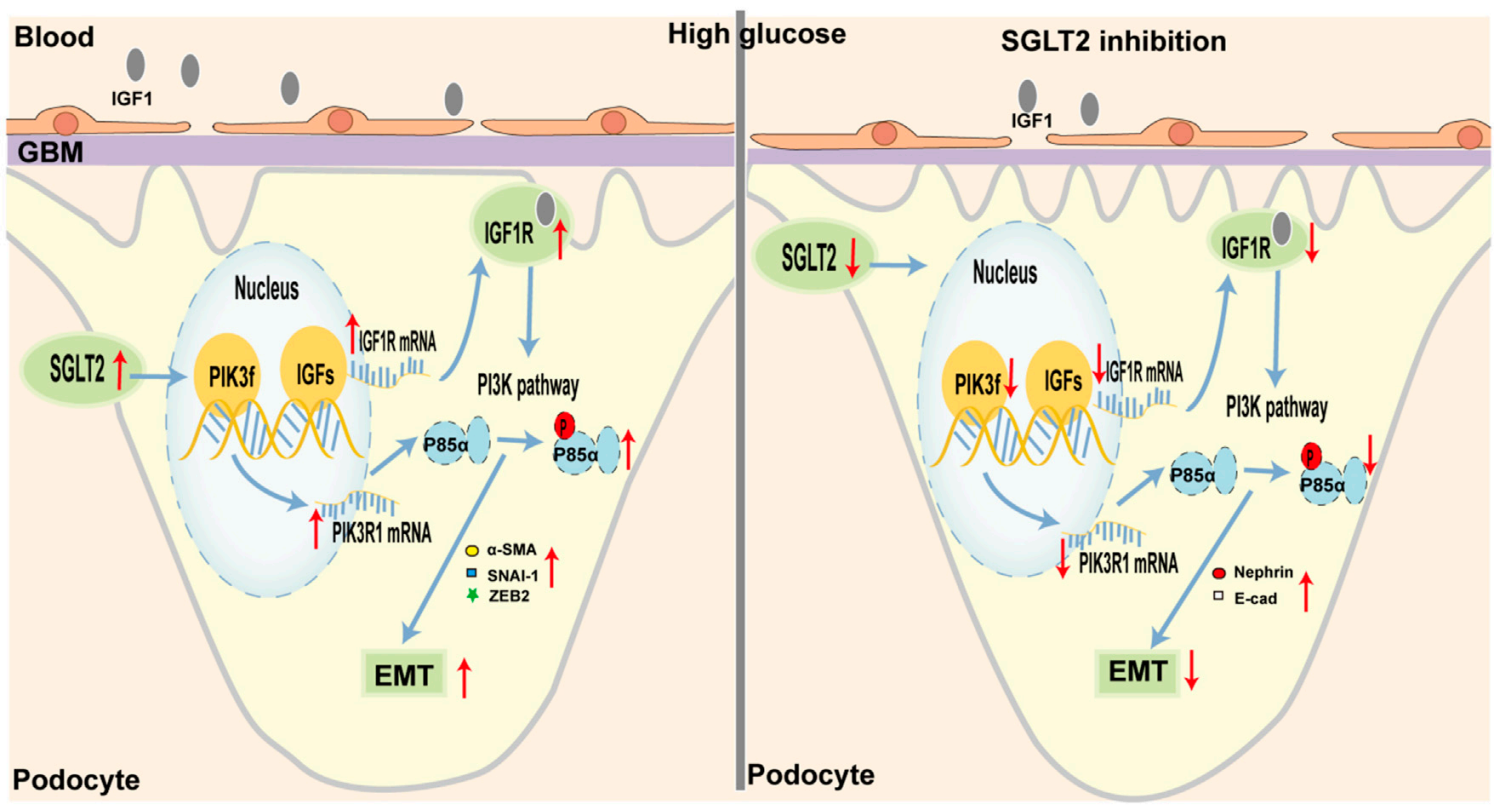
Schematic representation of SGLT2/IGF1R/PI3K-regulated EMT in podocytes under diabetic conditions. (Left) In diabetes, glycemic dysregulation caused the overexpression of SGLT2 in podocytes, which then promoted the formation of an intracellular hyperglycemic microenvironment. In response to high-glucose stimulation, the transcriptional and translational capacities of IGF1R/PI3K signaling were enhanced. At the molecular level, IGF1 and IGF2 expression was increased and closely bound to IGF1 receptors, further activated the phosphorylation of PI3K, and finally led to podocyte dysfunction, such as enhanced EMT. Microscopically, podocyte loss or fusion and glomerular fibrosis occurred. Macroscopically, proteinuria appeared. (Right) When SGLT2 was inhibited, podocyte damage mediated by the IGF1R/PI3K signaling pathway was inhibited, such as decreased IGF1 and IGF2 circulating levels, and increased nephrin and E-cad.

In our study, we provided substantial evidence to verify that SGLT2-is limited podocyte EMT by decreasing IGF1R/PI3K activity in *in vitro* and *in vivo* experiments. To some extent, the finding helped explain the anti-proteinuria effect of SGLT2-is, which suggested that podocytes might also be a major target of SGLT2 (Figure 6). Considering the specific expression of SGLT2 in the proximal tubules, previous studies about DN mainly focused on its beneficial effects by maintaining normal glycemia, thereby delaying DN progression (Kim et al., 2021; Sen et al., 2022). Several studies recently reported a direct role of SGLT2 in interfering with podocyte dysfunction. For instance, in mice with DN, the reno-protective effect of empagliflozin against proteinuria was primarily due to the decreased release of substances that damaged endothelial cells after SGLT2 downregulation in podocytes (Locatelli et al., 2022). Particularly, Cassis et al. (2018) reported increased SGLT2 expression in the HPC of proteinuric CKD patients as well as podocytes cultured with repeated BSA exposure. In support of this observation, our experiments found that intervention with dapagliflozin could limit podocyte SGLT2 upregulation and EMT-induced damage in a dose-dependent manner. As we all know, hyperglycemia is the major contributor to DN, which is clinically characterized by proteinuria (DeFronzo et al., 2021). Although SGLT2 inhibitors, which are well-recognized glucose-lowering agents, are involved in improving every pathological aspect of DN (Gnudi et al., 2016), more studies are needed to better verify the direct effect of SGLT2 inhibition on improving podocyte function.

Another important finding was that dapagliflozin modulated the EMT of podocytes *via* the IGF1R/PI3K pathway, which provided a conceivable connection between SGLT2 inhibition and reduced proteinuria. In dapagliflozin-treated DN mice or high-glucose-stimulated podocytes, the mRNA and protein expression profiles in the isolated renal cortex showed downregulation of IGF1R. IGF1R binds with high affinity to IGF1, followed by IGF2, which is activated to promote cellular glucose uptake and utilization by cells (Li et al., 2018). The compensatory upregulation of IGF1R in the early stage of DN promotes the energy use of target cells, while long-term excessive increases in IGF1R expression result in metabolic damage. For example, aminoguanidine, which inhibits glycosylation, was reported to decrease the renal expression of IGF1 and further improve mesangial expansion (Lupia et al., 1999; Peiris et al., 2017). In STZ-induced diabetic mice, activation of IGF-1 promoted renal fibrosis by upregulating SNAI-1 (Dong et al., 2019). When translating this observation to the *in vitro* experiments, we observed a marked upregulation of IGF1R and EMT markers, such as α-SMA, SNAI-1, and ZEB2, in cultured HPC exposed to high-glucose concentrations. This effect was not observed in HK-2, HMC, or HGREC, which suggested that the SGLT2/IGF1R axis functioned mainly in podocytes. In particular, when SGLT2 or IGF1R was inhibited, the effects on downstream factors and EMT markers were all gradually alleviated. A consistent previous study demonstrated that the IGF system helped to maintain the integrity of podocytes and glomeruli. Histological analysis of IGF1 transgenic mice showed obvious nuclear condense and foot process fusion (Bridgewater et al., 2008). Another study revealed that IGF-binding protein-3 enhanced TGF-β-mediated apoptosis and decreased BM7-induced anti-apoptotic signaling, which altogether resulted in podocyte loss (Peters et al., 2006). The insulin/IGF1R signaling pathway could promote mitochondrial fusion in podocytes, which further affected energy utilization (Ising et al., 2015). Thus, it is reasonable that we hypothesized that the high expression of SGLT2 in podocytes could promote the transition to the epithelium and become dysfunctional and thus contributed to and aggravated proteinuria.

Moreover, among the various molecular mechanisms regulated by IGF1R, the most common mechanism is the autophosphorylation of downstream protein kinases, which triggers signal transduction and results in disease development (Peiris et al., 2017; Qiao et al., 2021). For instance, the PI3K/AKT/mTOR pathway, which is a classical pathway of IGF1R action, is activated to inhibit cell apoptosis and stimulate protein synthesis (Wang et al., 2020). Consistent with these studies, we also observed enhanced transcription and translation of PI3K in high-glucose-stimulated podocytes. EMT is a characteristic feature of podocyte damage. Xing et al. (2015) reported that the PTEN/PI3K/AKT pathway mediated EMT in high-glucose-stimulated podocytes, and inhibition of this pathway prevented phenotypic transition. PI3K/AKT phosphorylation is involved in the signal transduction of proinflammatory and fibrogenic factors, which affected podocyte homeostasis (Luo et al., 2021). In STZ-induced DN models, islet transplantation increased the expression of the podocyte marker, nephrin, and decreased the protein level of the mesenchymal marker, α-SMA (Guo et al., 2014). Consistent with these results, we observed the upregulation of α-SMA, SNAI-1, ZEB2 and the downregulation of nephrin and E-cad in both DN mice and high-glucose-stimulated podocytes. Consistent with this view, the KEGG analysis in our study also revealed enrichment of the phosphatidylinositol signaling system and insulin signaling pathway, which contributed to the EMT of podocytes (Zhang et al., 2019; Zheng et al., 2020).

SGLT2-is-treated DN patients exhibited decreased circulating levels of IGF1 and IGF2, possibly due to the reduction of its synthesis in the liver. In addition, the expression level of IGF2 was significantly higher than that of IGF1, and this suggested a possible role of IGF2 in mediating IGF1R activation. Several clinical studies have observed increased expression of IGF1 and IGF2 in patients with DN (Sireesha et al., 2009; Shao et al., 2016), but the central roles of the two ligands remain controversial and need further study. As IGF1R inhibitors have not entered the clinical trial stage yet, it is not feasible to assess the clinical effects of these inhibitors in patients with DN. In our *in vitro* trial, OSI-906 helped to stabilize podocyte integrity, in combination with dapagliflozin or not, which provided evidence for its protective role in DN.

In summary, through mRNA-seq, we discovered the anti-EMT effect of IGF1R/PI3K signaling in dapagliflozin-treated DN mice. We inferred that enhanced expression of components of the IGF1R/PI3K pathway could accelerate the EMT of podocytes, ultimately leading to progressive proteinuria. Our study supported the use of IGF1R inhibitors in reducing glomerular proteinuria.

## Data Availability

.The datasets presented in this study can be found in https://www.ncbi.nlm, and the accession number is GSE200322.

## Supplementary material

Supplementary Figure S1 Representative IHC images of SGLT2 expression in glomeruli from the Con, DN, and DA groups.

Supplementary Figure S2 Representative images of HE, PAS, Masson staining, and IHC of collagen IV in glomeruli from the Con, DN, and DA groups.

Supplementary Figure S3 Representative electron micrographs of Con, DN, and DA mice. Red arrow: thickened GBM and damaged podocyte.

Supplementary Figure S4 Venn diagram of all 1957 DEGs. DEGs: differentially expressed genes.

Supplementary Figure S5 Heatmap of all 1957 DEGs. Red: upregulated; green: downregulated.

Supplementary Figure S6 IGF1R-centered PPI network (combined score >0.4).

Supplementary Figure S7 IGF system and EMT-related DEGs among the three groups.

Supplementary Figure S8 Representative IHC images of IGF1 and IGF1R expression in glomeruli from the Con, DN, and DA groups.

Supplementary Figure S9 IGF1R expression levels in cultured HMC **(A)**, HGREC **(B)**, HK-2 cells **(C)**, and HPC **(D)**. HMC: human mesangial cell; HGREC: human glomerular endothelial cell; HK-2: human tubular epithelial cell line; HPC: human podocytes; Man: mannitol-treated group (34.4 mM). Data were analyzed by ANOVA followed by Tukey’s post hoc test. N=3; * P<0.05, **P<0.01, and * P<0.05, ns: no significance.

## Nomenclature

DM: diabetic mellitus
DN: diabetic nephropathy
SGLT2: sodium–glucose cotransporter 2
SGLT2-is: inhibitors of SGLT2
IGF1: insulin-like growth factor-1
IGF2: insulin-like growth factor-2
IGF1R: IGF1 receptor
IGFs: IGF system
EMT: epithelial–mesenchymal transition
HPC: human podocytes
HRGEC: human renal glomerular endothelial cell
HMC: human mesangial cell
HK-2: human tubular epithelial cell line
DA: dapagliflozin
OSI-906: inhibitor of IGF1R
STZ: streptozotocin
BW: body weight
BG: blood glucose
SCr: serum creatinine
PCR: protein and creatinine ratio
BUN: blood urea nitrogen
IHC: immunohistochemical staining
IF: immunofluorescence
PCA: principal component analysis
KEGG: Kyoto Encyclopedia of Genes and Genomes
ANOVA: analysis of variance
DEG: differentially expressed genes
FPKM: fragments per kilobase of exon per million reads
PPI: protein–protein interaction
