# Corrigendum: SGLT2 inhibitors suppress epithelial-mesenchymal transition in podocytes under diabetic conditions *via* downregulating the IGF1R/PI3K pathway

**DOI:** 10.3389/fphar.2022.1074294

**Published:** 2022-12-22

**Authors:** Ruixue Guo, Peipei Wang, Xuejun Zheng, Wen Cui, Jin Shang, Zhanzheng Zhao

**Affiliations:** ^1^ Department of Nephrology, The First Affiliated Hospital of Zhengzhou University, Zhengzhou, China; ^2^ Zhengzhou University, Zhengzhou, China; ^3^ Nephropathy Laboratory, The First Affiliated Hospital of Zhengzhou University, Zhengzhou, China; ^4^ Laboratory Animal Platform of Academy of Medical Sciences, Zhengzhou, China

**Keywords:** diabetic nephropathy, sodium-glucose cotransporter-2 inhibitors, insulin-like growth factor-1 receptor, podocyte, epithelial-mesenchymal transition

In the original article, there was a mistake in Figure 1C, **Supplementary Figure S2**, Figure 3B, and Figure 4 as published:

(1) In Figure 1C and **Supplementary Figure S2**, the IHC images of IGF1R were erroneously used as the results of Collagen IV. The corrected IHC images of Collagen IV were displayed as below.
(2) In Figure 3B, WB gels related to SGLT2 and IGF1R should be annotated according to Con-DA-DN. The corrected Figure 3B was displayed as below.
(3) In Figure 4A, the annotation of WB gel for Nephrin and α-SMA was reversed. The corrected Figure 4A was displayed as below.
(4) In Figures 4B,D, the annotation of the bar for Nephrin and α-SMA was reversed. The corrected Figure 4 was displayed as below.
(5) **Supplemenary Figures S10–S12** are added as the raw data of Figures 3, 4 and **Supplemenary Figure S9**.

**FIGURE 1:**
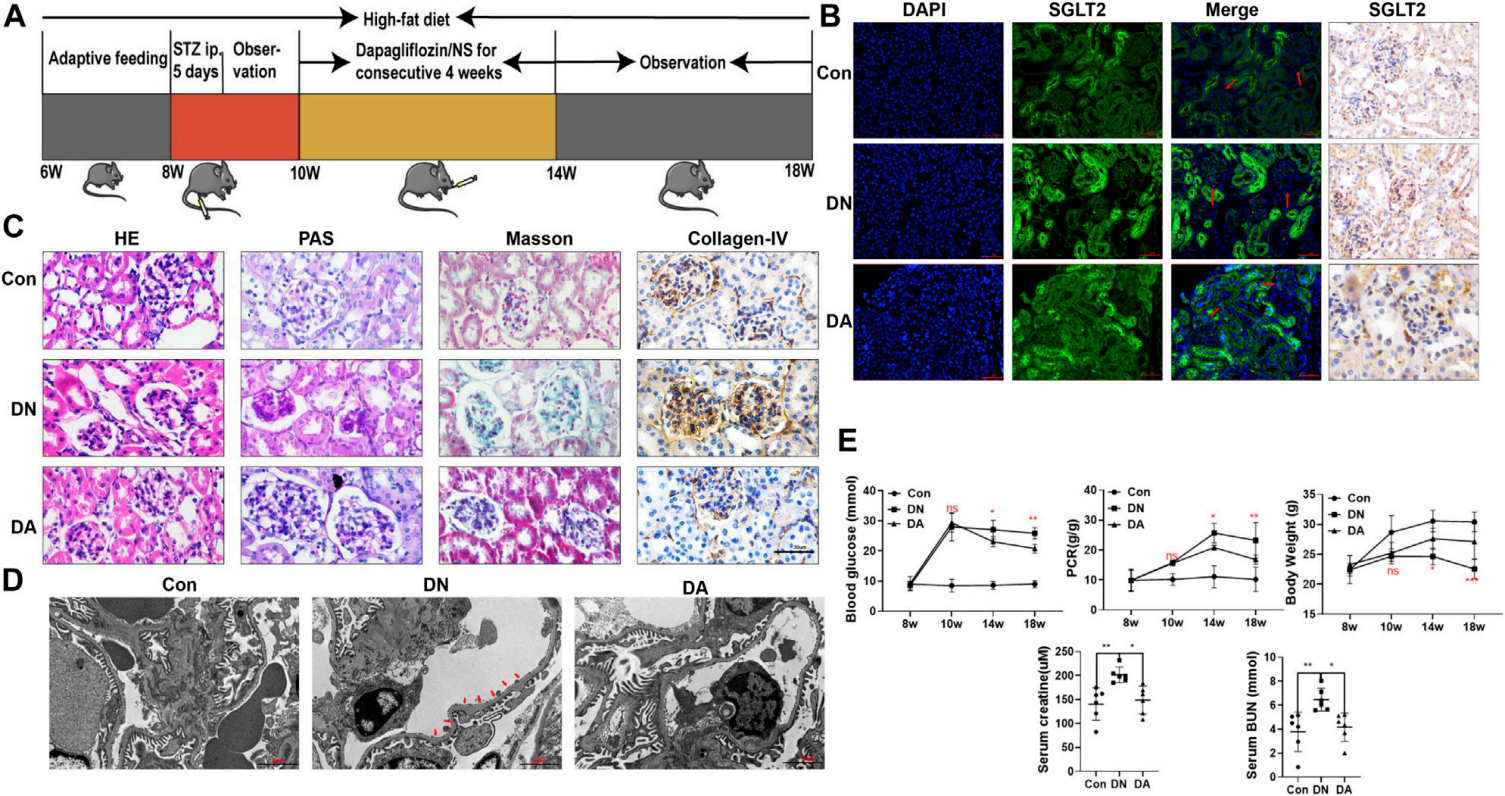
Dapagliflozin attenuated STZ-induced diabetic kidney injury. **(A)** Flowchart of the animal experiment (*n* = 6/group). **(B)** IF and IHC staining of SGLT2. **(C)** Pathological staining: light micrographs of HE, PAS, and Masson staining and IHC of collagen IV. **(D)** Representative electron micrographs of glomeruli. Areas of basement membrane thickening and podocyte injury are indicated by red arrows. **(E)** Statistical significance of BG levels, BW, and PCR among the three groups in the 8th, 10th, 14th, and 18th weeks was performed using two-way ANOVA, and Tukey’s algorithm for subsequent multiple comparisons between two groups. In parallel, one-way ANOVA was performed for SCr and BUN levels. STZ: streptozotocin; Con: control group; DN: diabetic nephropathy; DA: dapagliflozin-treated DN group: NS: normal saline; SGLT2: sodium–glucose cotransporter 2; IF: immunofluorescent staining; IHC: immunohistochemical staining; one-way ANOVA: one-way analysis of variance; BG: blood glucose; BW: body weight; PCR: urinary total protein to creatinine ratio; SCr: serum creatinine; BUN: blood urea nitrogen.

**FIGURE 3:**
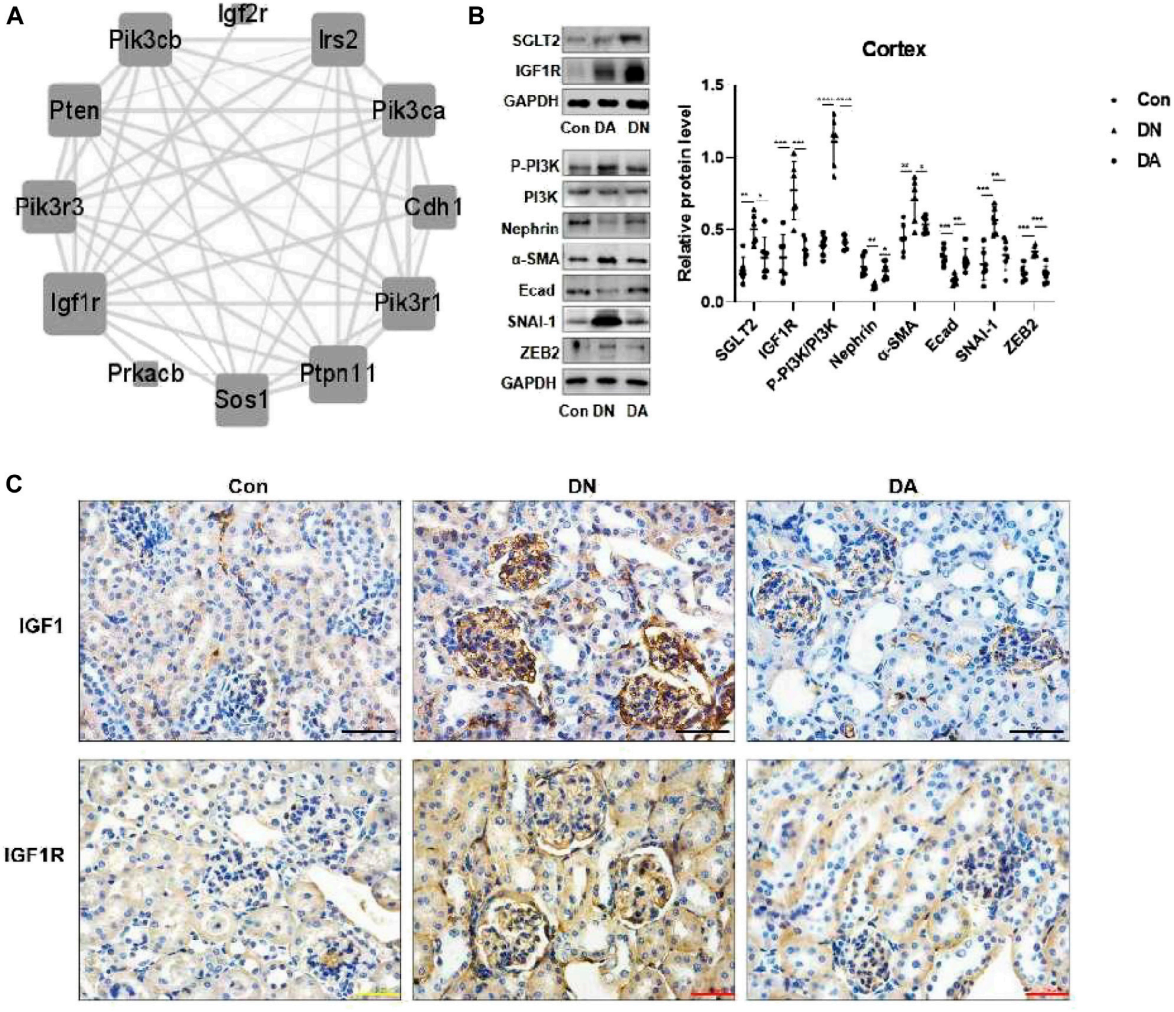
In DN models, upregulation of SGLT2 activated IGF1R/PI3K signaling. **(A)** IGF1R-centered PPI network (combined score>0.9). **(B)** Protein expression levels of SGLT2, IGF1R, phosphorylated PI3K, and EMT markers were analyzed using Western blotting. The data were analyzed by ANOVA with Tukey’s post hoc test. **(C)** IHC images of IGF1 and IGF1R. PPI: protein–protein interaction; *p < 0.05, **p < 0.01.

**FIGURE 4:**
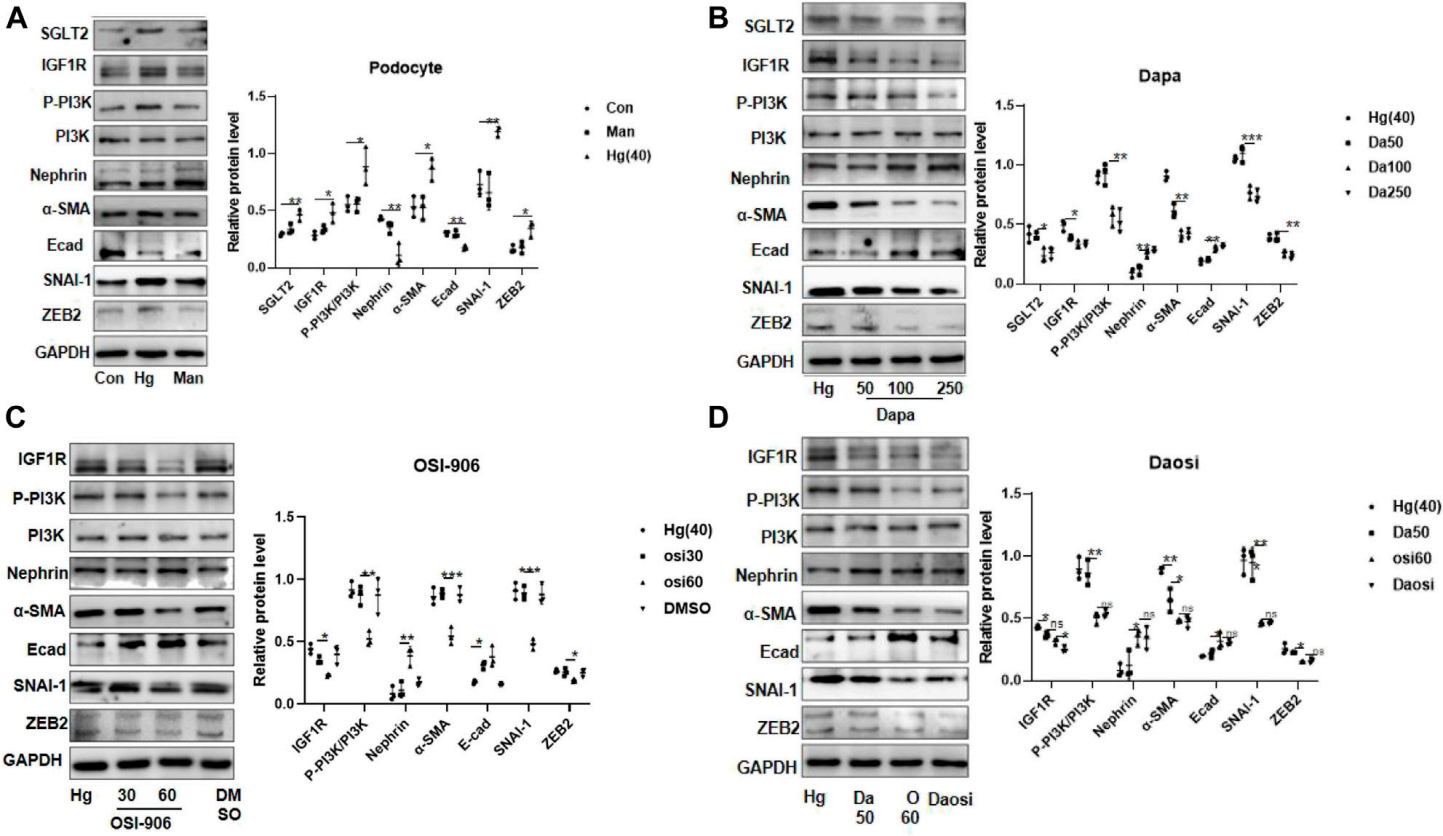
SGLT2 directly regulated podocyte EMT *via* IGF1R/PI3K signaling. **(A)** Representative WB images and densitometric analysis of the expression of SGLT2, IGF1R, phosphorylated PI3K, and EMT markers in high-glucose-stimulated podocytes (Hg: 40 nM, 24 h). **(B)** Podocytes treated with different doses of dapagliflozin (50 nM, 100 nM, and 250 nM) and **(C)** OSI-906 (30 nM and 60 nM). **(D)** Combination of OSI60 and Da50. EMT: epithelial–mesenchymal transition. The data were analyzed by ANOVA with Tukey’s post hoc test. N = 3; *p < 0.05, **p < 0.01, ***p < 0.001, ****p < 0.001.

The authors apologize for this error and state that this does not change the scientific conclusions of the article in any way. The original article has been updated.

