# Supplementary figures and images for: SGLT2 inhibitors suppress epithelial–mesenchymal transition in podocytes under diabetic conditions via downregulating the IGF1R/PI3K pathway
Source: Front Pharmacol. 2022 Sep 26;13:897167. doi: 10.3389/fphar.2022.897167 (PMC9550168; doi:10.3389/fphar.2022.897167)

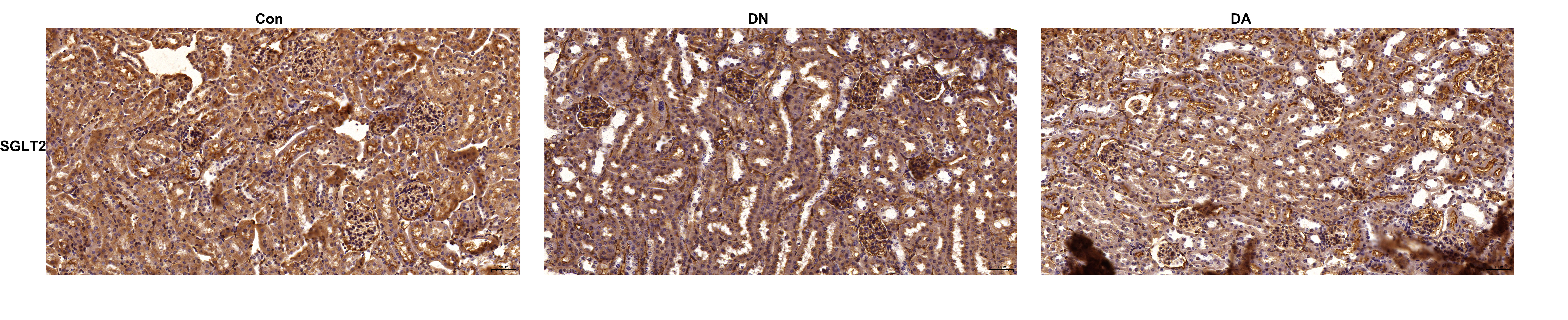

Supplement: Supplementary file 1 [file DataSheet1.zip › Supplementary file/Supplementary figure 1.jpg]

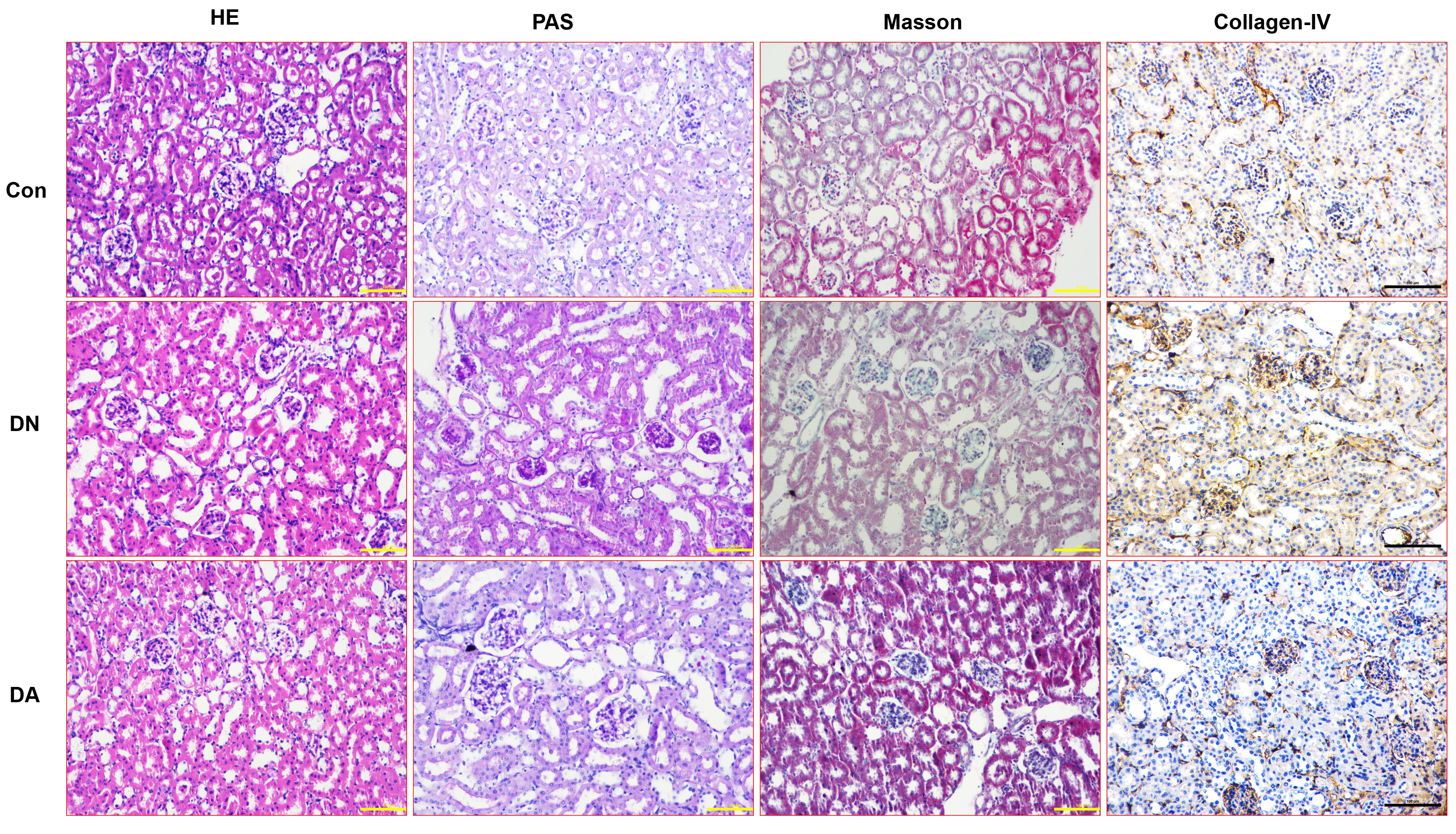

Supplement: Supplementary file 1 [file DataSheet1.zip › Supplementary file/Supplementary figure 2.jpg]

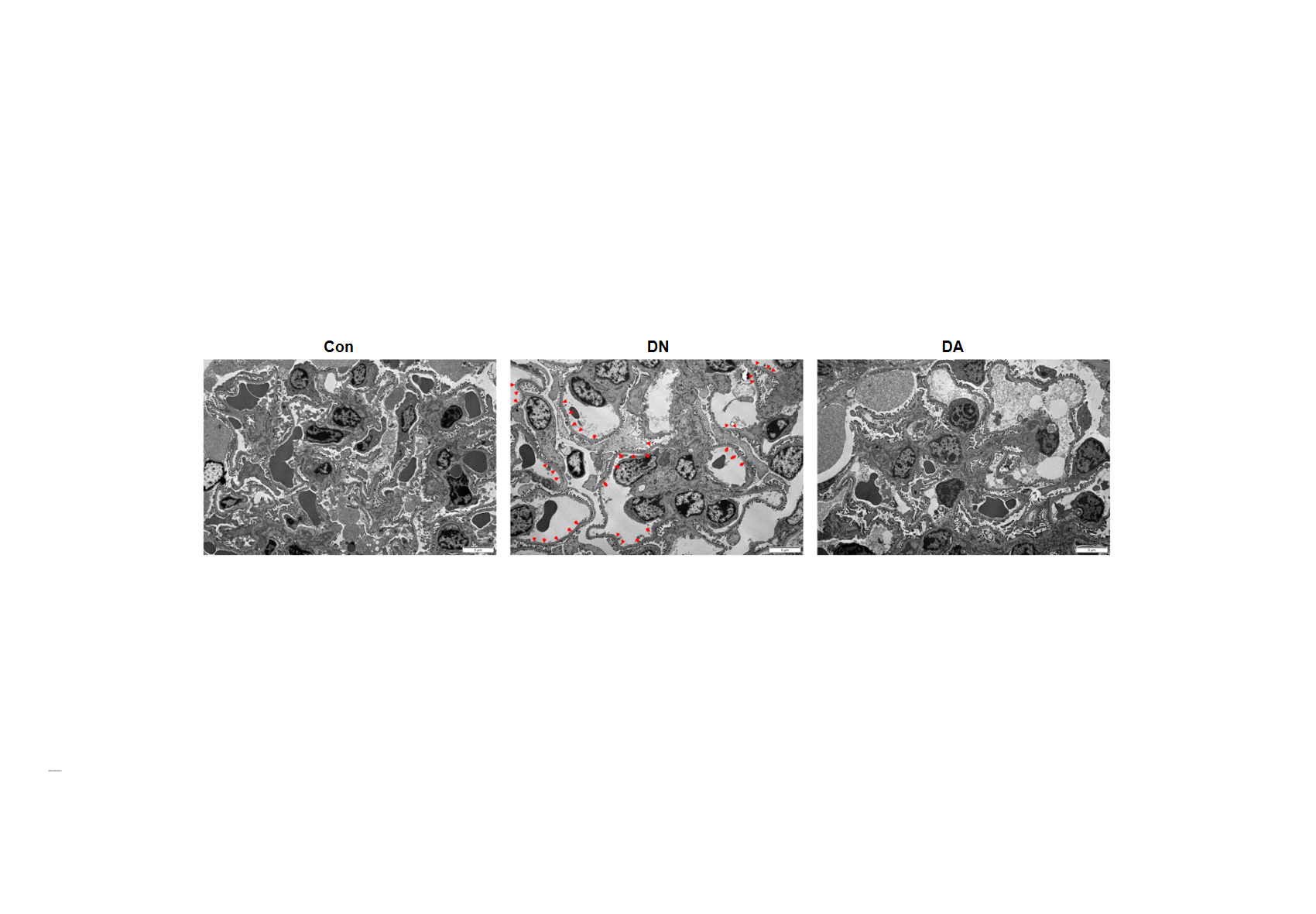

Supplement: Supplementary file 1 [file DataSheet1.zip › Supplementary file/Supplementary figure 3.tif]

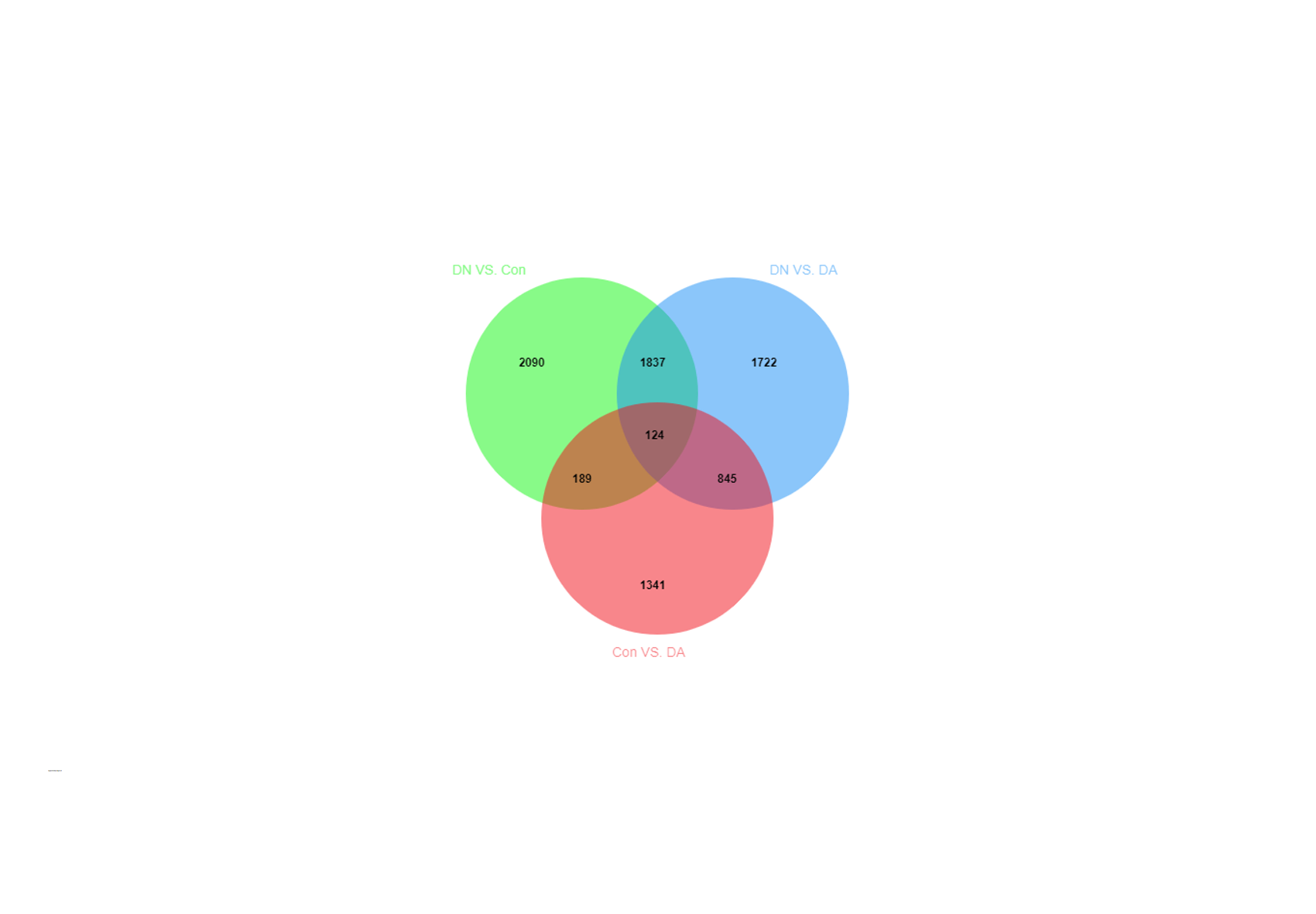

Supplement: Supplementary file 1 [file DataSheet1.zip › Supplementary file/Supplementary figure 4.tif]

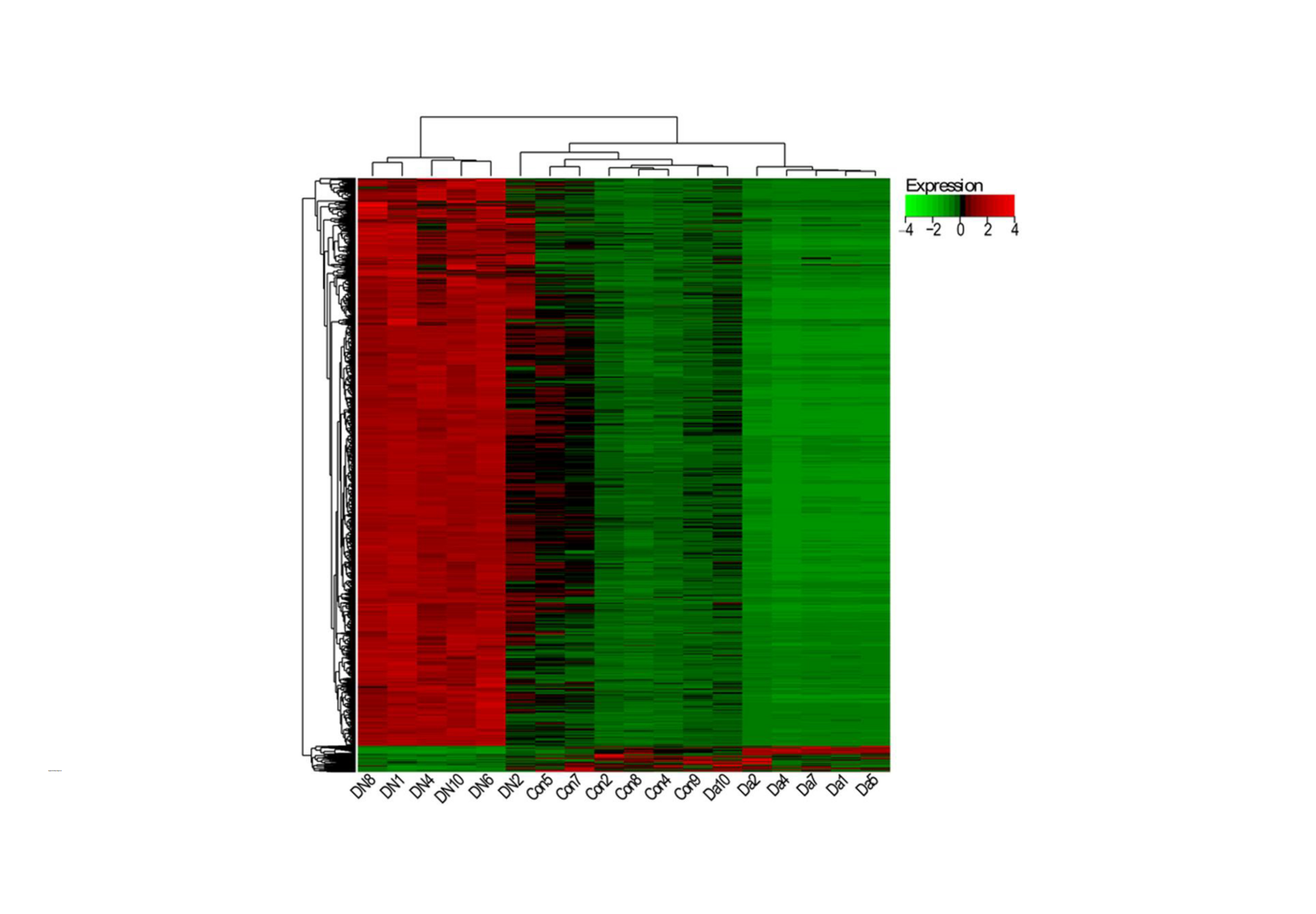

Supplement: Supplementary file 1 [file DataSheet1.zip › Supplementary file/Supplementary figure 5.tif]

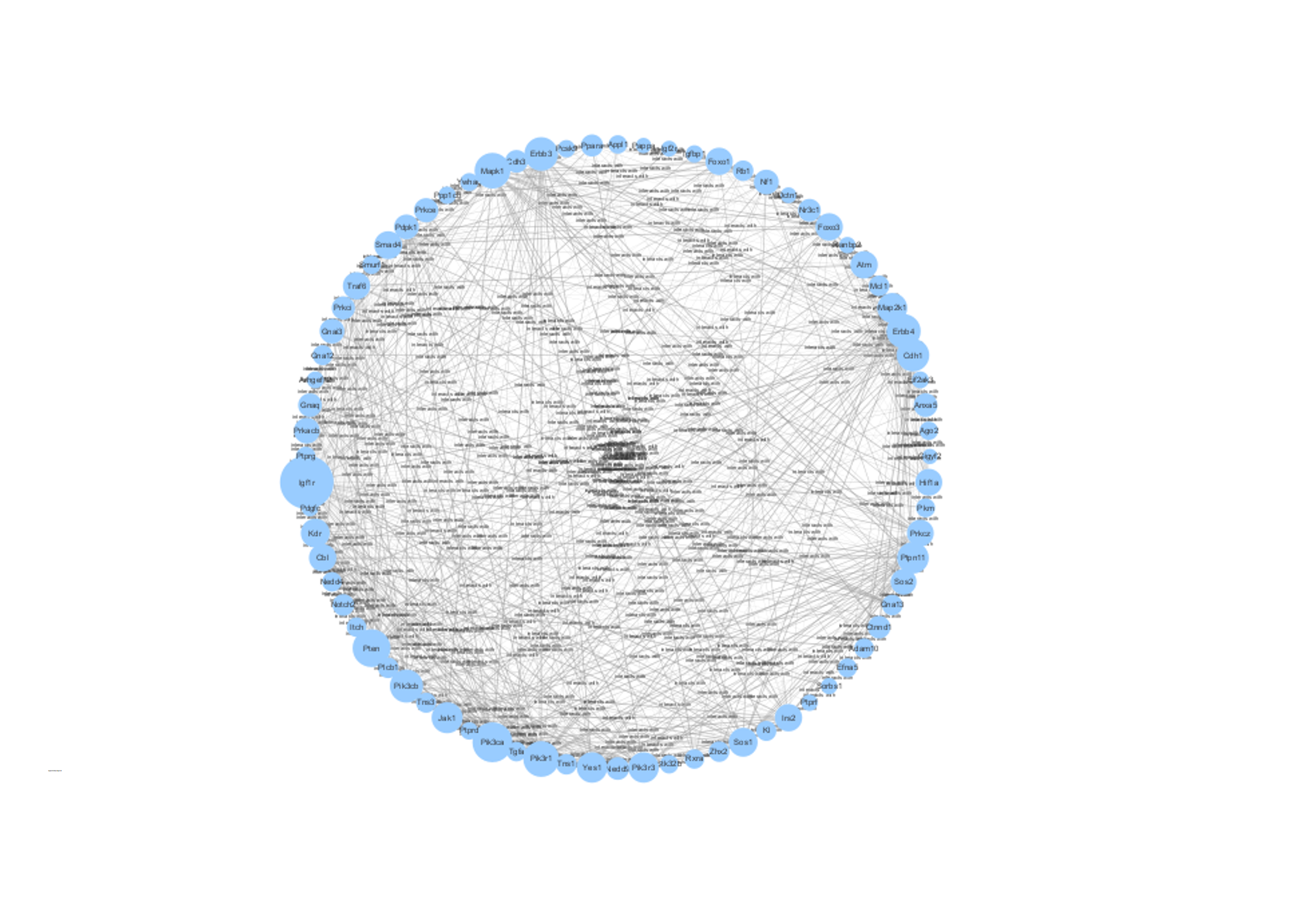

Supplement: Supplementary file 1 [file DataSheet1.zip › Supplementary file/Supplementary figure 6.tif]

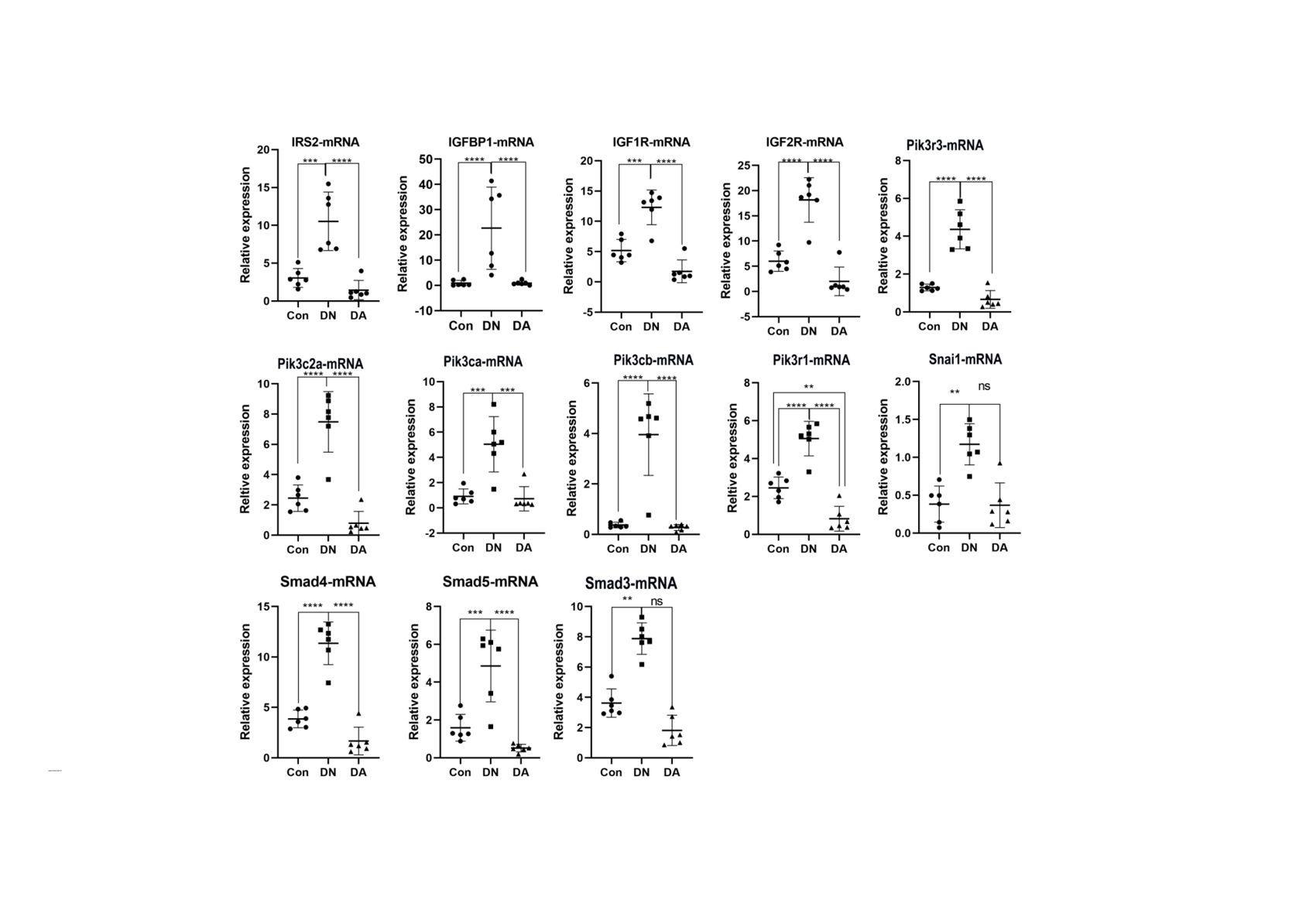

Supplement: Supplementary file 1 [file DataSheet1.zip › Supplementary file/Supplementary figure 7.tif]

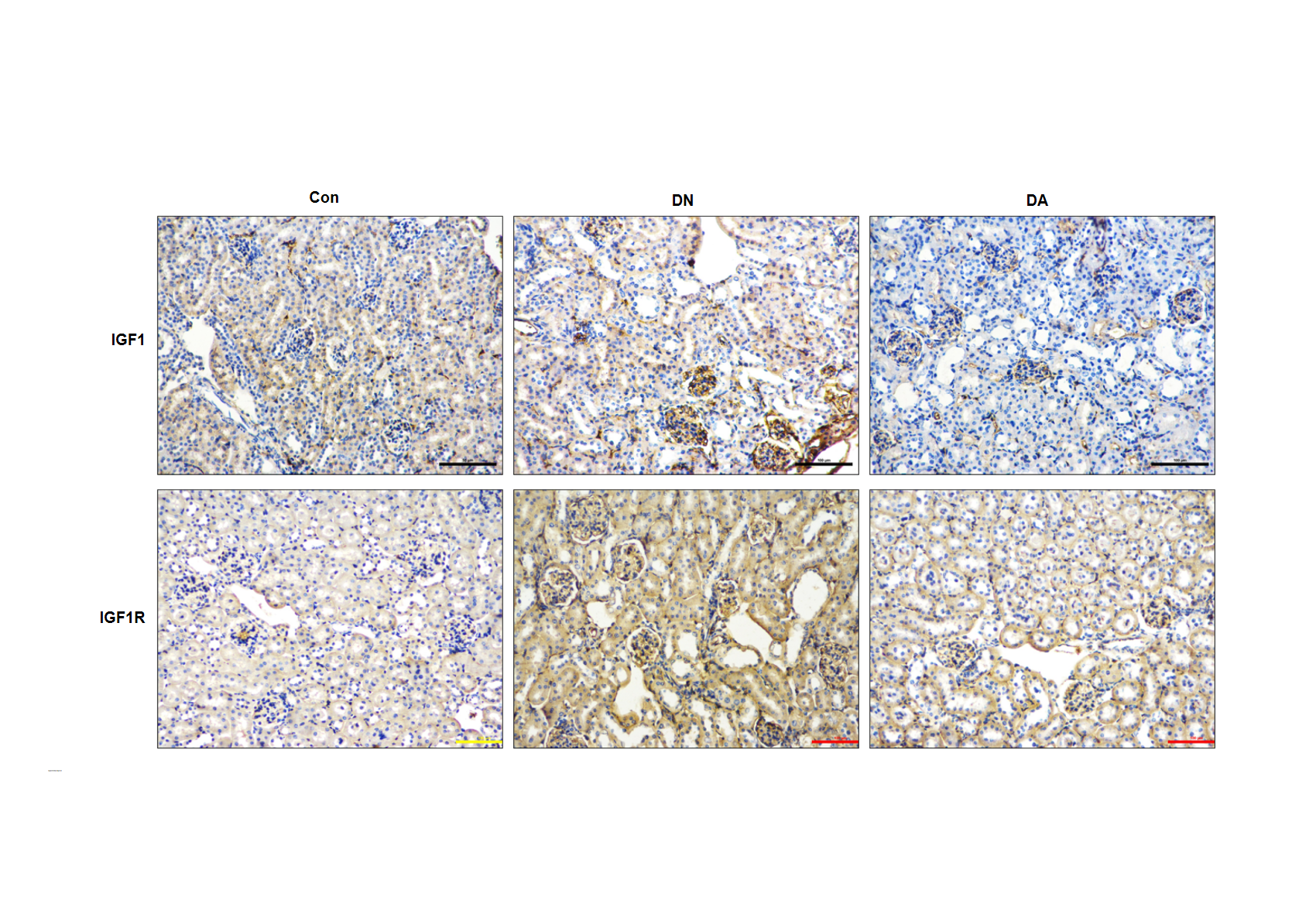

Supplement: Supplementary file 1 [file DataSheet1.zip › Supplementary file/Supplementary figure 8.tif]

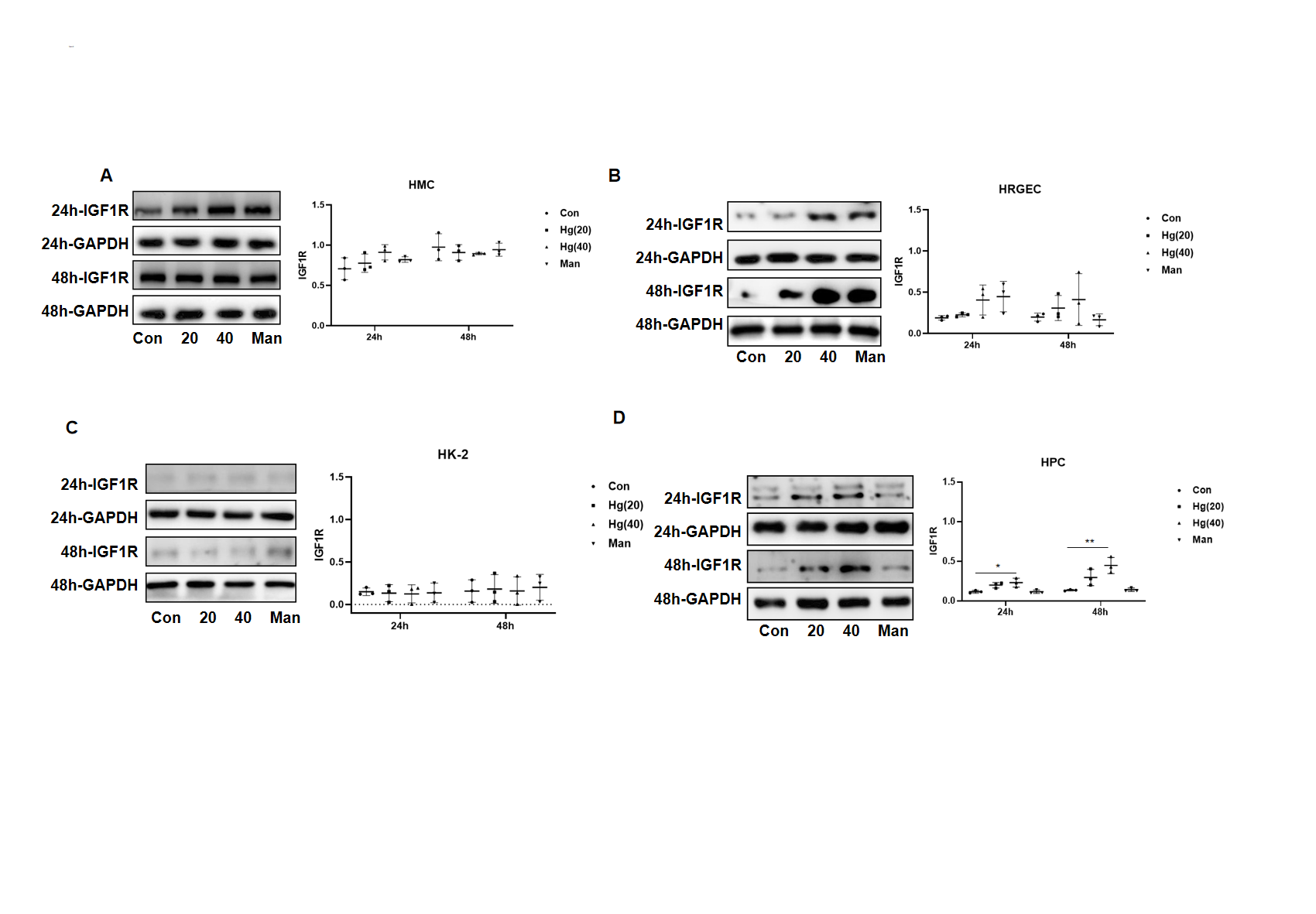

Supplement: Supplementary file 1 [file DataSheet1.zip › Supplementary file/Supplementary figure 9.tif]

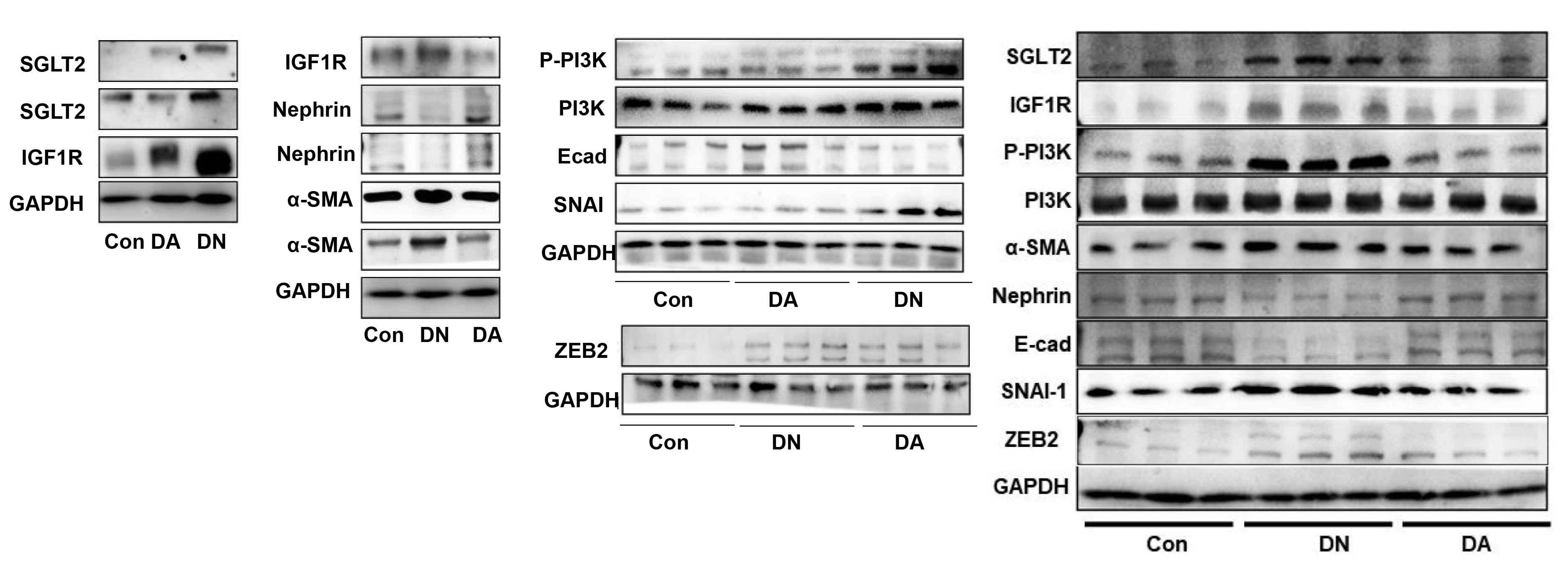

Supplement: Supplementary file 1 [file DataSheet1.zip › Supplementary file/Supplementary figure s10.jpg]

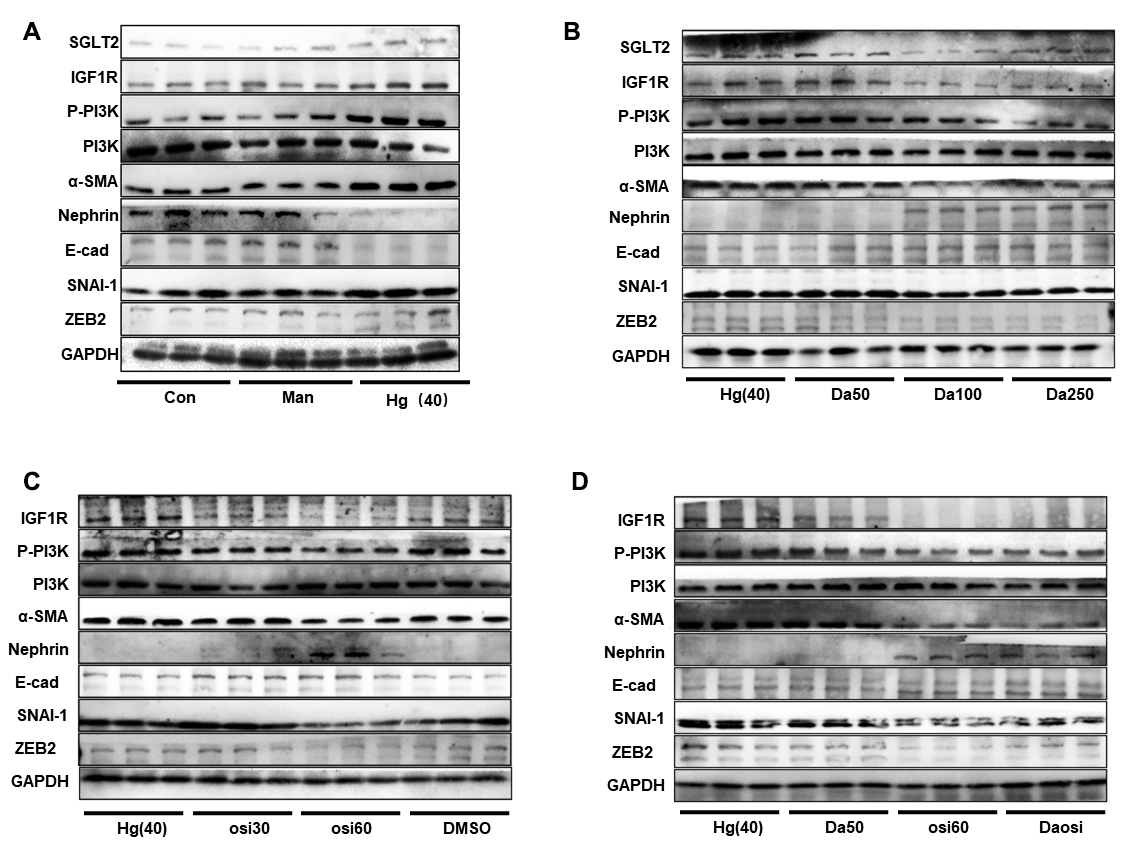

Supplement: Supplementary file 1 [file DataSheet1.zip › Supplementary file/Supplementary figure s11.jpg]

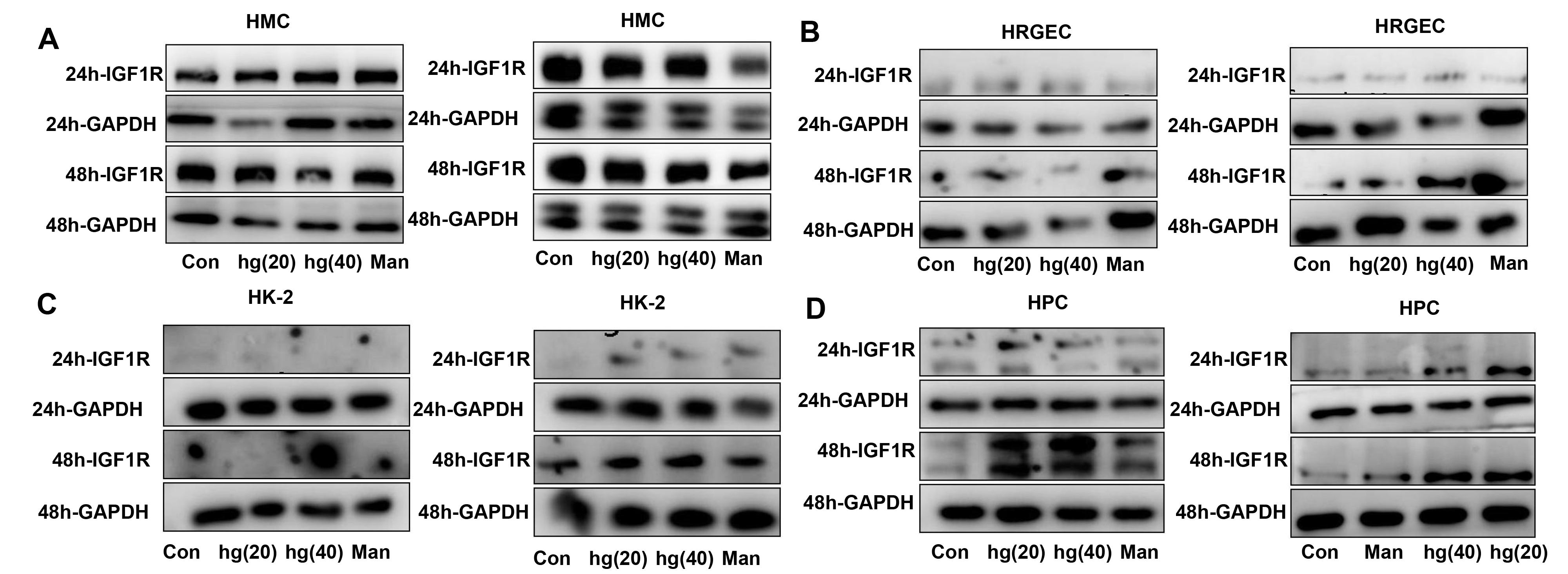

Supplement: Supplementary file 1 [file DataSheet1.zip › Supplementary file/Supplementary figure s12.jpg]
